# Protocol to measure protein-RNA binding using double filter-binding assays followed by phosphorimaging or high-throughput sequencing

**DOI:** 10.1016/j.xpro.2023.102336

**Published:** 2023-06-03

**Authors:** Joel Vega-Badillo, Phillip D. Zamore, Karina Jouravleva

**Affiliations:** 1 RNA Therapeutics Institute, University of Massachusetts Chan Medical School, 368 Plantation Street, Worcester, MA 01605, USA; 2 Howard Hughes Medical Institute, University of Massachusetts Chan Medical School, 368 Plantation Street, Worcester, MA 01605, USA

**Keywords:** Molecular Biology, Protein Biochemistry

## Abstract

Binding affinity quantitatively describes the strength of a molecular interaction and is reported by the equilibrium dissociation constant (*K*_D_). Here, we present a protocol to measure *K*_D_ of mammalian microRNA-loaded Argonaute2 protein by double filter binding. We describe steps for radiolabeling target RNA, measuring concentration of binding-competent protein, setting up binding reactions, separating protein-bound RNA from protein-unbound RNA, preparing library for Illumina sequencing, and performing data analysis. Our protocol is easily applied to other RNA- or DNA-binding proteins.

For complete details on the use and execution of this protocol, please refer to Jouravleva et al.^1^

## Highlights

• A single-step binding assay that uses a simple experimental setup
• Double filter-binding assays quantify concentration of active protein
• Double filter-binding assays measure the affinity of a protein for a target RNA
• Readily adapted to high-throughput measurements by RNA Bind-n-Seq

## Abstract (editor highlights)

Publisher’s note: Undertaking any experimental protocol requires adherence to local institutional guidelines for laboratory safety and ethics.

## Graphical abstract

**Figure:**
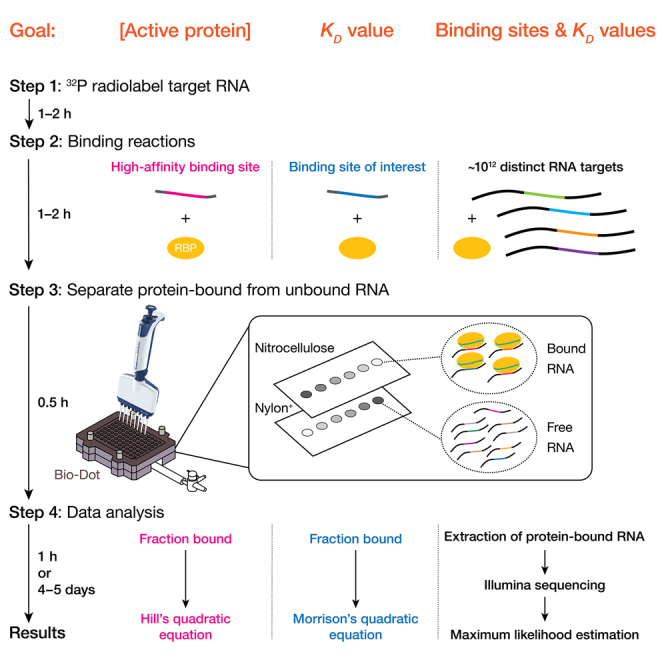

## Before you begin

A variety of methods measure the binding affinity of an RNA-binding protein (RBP): e.g., electrophoretic mobility shift assays,^2^ isothermal titration calorimetry,^3^ fluorescence anisotropy,^4^ and double filter-binding assay.^5^ Among these, the double filter-binding assay uses a simple, inexpensive experimental setup and is readily adapted to high-throughput measurements.^1^^,^^6^ The protocol below describes the specific steps for performing double filter-binding assays to measure the binding affinity of an RBP for a specific binding site. Double filter-binding assays yield results in a couple of hours but interrogate only known interactions and are therefore inherently low-throughput. However, when followed by high-throughput sequencing, double filter-binding enables de novo discovery of binding motifs and estimation of *K*_D_ values for dozens to hundreds of binding sites simultaneously. Because the maximum amount of bound ligand is determined by the concentration of total active RBP, we also describe how to use double filter-binding assays to quantify the concentration of binding-competent protein. Purified RBP is a prerequisite for the assay; to illustrate the protocol, we use mouse RISC (RNA-induced silencing complex), comprising a purified Argonaute2 (AGO2) protein and a synthetic microRNA (miRNA) guide.

### Preparation of RISC

**Timing: 5−6 days**

The procedure below describes preparation of mouse miR-449a·AGO2 RISC.

***Note:*** A double filter-binding assay for a single RNA target typically requires ∼20 fmol RISC; for a single RNA Bind-n-Seq (RBNS) assay ∼170 fmol RISC is needed.

1. Grow and expand HEK293T cells that stably overexpress mouse AGO2 protein bearing an N-terminal 3XFLAG tag.^1^

**Figure 1:**
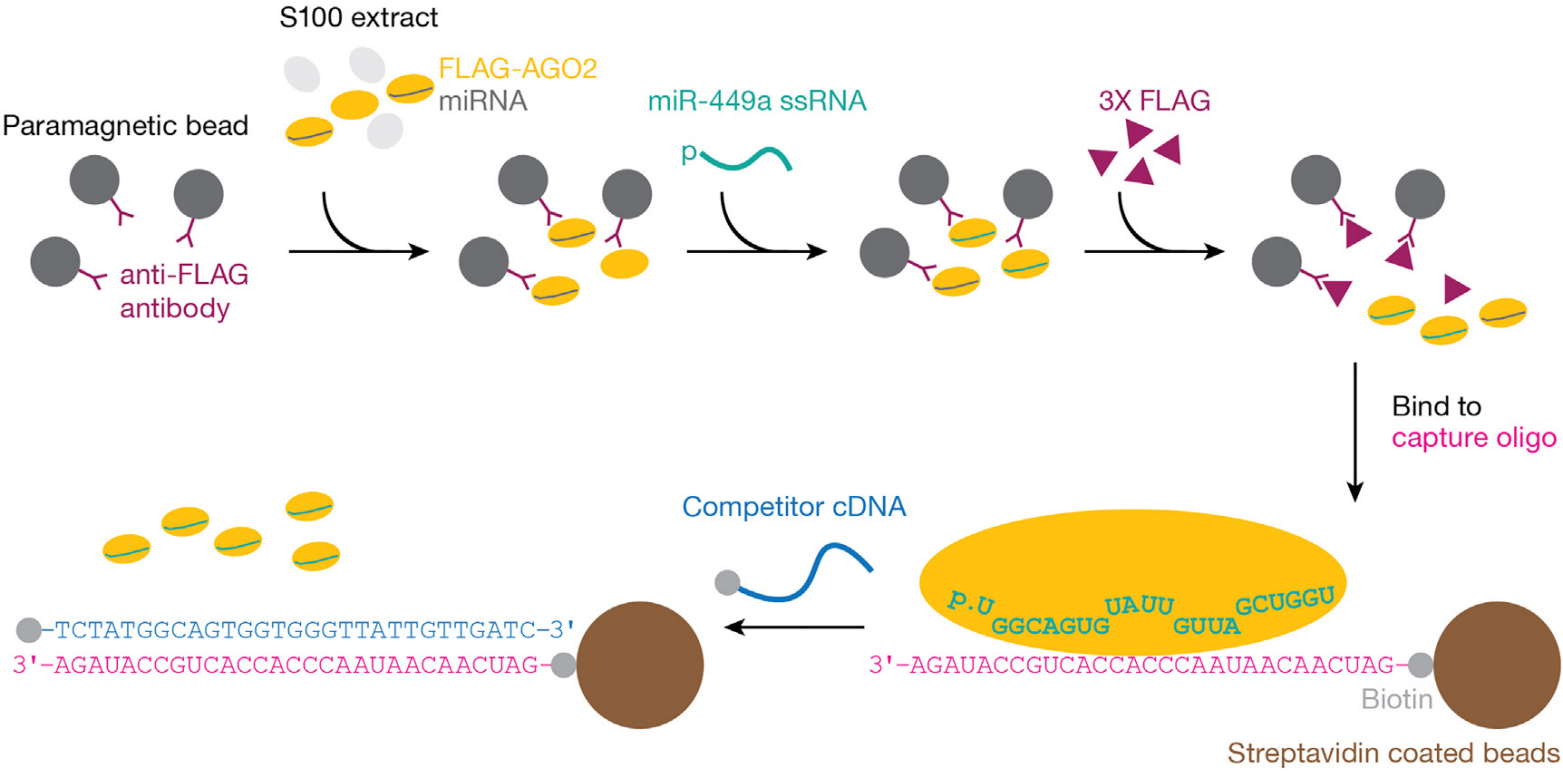
Strategy to purify active RISC

***Note:*** Typically, five 95%-confluent 100-cm^2^ culture dishes of these cells yield 100 fmol of miRNA-loaded, purified mouse AGO2.

2. Prepare cytosolic S100 extracts according to ref.^7^
  a. Prepare Buffer A, Buffer B, and Buffer C. Buffers without DTT can be stored at room temperature (20°C−25°C) up to 3 months.

**Table:** Buffer A

Reagent	Final concentration	Amount
HEPES-KOH, pH 7.9 (1 M)	10 mM	2.5 mL
Potassium acetate (2.5 M)	10 mM	1 mL
Magnesium acetate (1 M)	1.5 mM	375 μL
DTT (1 M)	0.5 mM	125 μL
CHAPS (1% (w/v))	0.01% (w/v)	2.5 mL
Nuclease-free water	N/A	243.5 mL
Total	N/A	250 mL

**Table:** Buffer B

Reagent	Final concentration	Amount
HEPES-KOH, pH 7.9 (1 M)	300 mM	75 mL
Potassium acetate (2.5 M)	1.4 M	140 mL
Magnesium acetate (1 M)	30 mM	7.5 mL
DTT (1 M)	0.5 mM	125 μL
CHAPS (1% (w/v))	0.01% (w/v)	2.5 mL
Nuclease-free water	N/A	24.9 mL
Total	N/A	250 mL
    **Table:** Buffer C

Reagent	Final concentration	Amount
HEPES-KOH, pH 7.9 (1 M)	30 mM	1.5 mL
Potassium acetate (2.5 M)	120 mM	2.4 mL
Magnesium acetate (1 M)	3.5 mM	175 μL
DTT (1 M)	2 mM	100 μL
CHAPS (1% (w/v))	0.01% (w/v)	0.5 mL
Glycerol	80% (w/v)	40 g
Nuclease-free water	N/A	to 50 mL
Total	N/A	50 mL
  b. Prepare 100× Protein Inhibitor Cocktail.

**Table:** 

Reagent	Final concentration	Amount
AEBSF	120 mg/mL	1.2 g
Aprotinin	1 mg/mL	0.01 g
Bestatin	7 mg/mL	0.07 g
E-64	1.8 mg/mL	0.018 g
Leupeptin	2.4 mg/mL	0.024 g
Nuclease-free water	N/A	to 10 mL
Total	N/A	10 mL

For immediate use, store the solution at 4°C. For long-term storage (up to 6 months), make 0.5-mL aliquots and keep at −20°C. Alternatively, EDTA-free Protein Inhibitor Cocktail can be purchased ready-to-use.
  c. Harvest cells.
    i. Pour off cell media from a cell dish into waste pot.
    ii. Add ∼5 mL ice-cold PBS onto the plate to cover the cell layer.
    iii. Using a cell scraper, gently scrape the cells off the bottom of the plate into PBS.
    iv. Transfer the detached cells into a 50-mL tube.
    v. Repeat for the all cell dishes, collecting cells in the same tube.
  d. Centrifuge cell at 500 × *g* for 5 min at 4°C.
  e. Wash cell pellets once with ice-cold PBS.
    i. Remove the supernatant from the previous step.
    ii. Add ∼50 mL ice-cold PBS.
    iii. Centrifuge at 500 × *g* for 5 min at 4°C.
  f. Remove all the supernatant and proceed to the next step. Alternatively, dry cell pellets can be snap frozen in liquid nitrogen and stored at –80°C until ready for the next step.
  g. Resuspend the cell pellet in twice its volume with Buffer A supplemented with Proteinase Inhibitor Cocktail.
  h. Incubate on ice for 20 min to allow the cells to swell.
  i. Lyse cells on ice with a Dounce homogenizer using 40 strokes of a tight pestle (B type).
  j. Centrifuge the homogenate at 2,000 × *g* for 10 min at 4°C to remove nuclei and cell membranes.
  k. Collect the supernatant and add 0.11 volumes (relative to the volume of the clarified supernatant) of Buffer B supplemented with Proteinase Inhibitor Cocktail. Mix by gentle inversion.
  l. Centrifuge at 100,000 × *g* for 20 min at 4°C. Collect the supernatant; the supernatant corresponds to the S100 extract.
  m. Add 0.32 volumes ice-cold Buffer C supplemented with Proteinase Inhibitor Cocktail to S100 to achieve a 20% (w/v) final glycerol concentration and mix by gentle inversion.
  n. Aliquot S100, flash-freeze in liquid nitrogen, and store at −80°C for up to 6 months.
3. Assemble RISC (Figure 1).
  a. Use 20 μL anti-FLAG M2 paramagnetic beads (Sigma) per mL S100.
  b. Briefly wash the beads three times with 1 mL ice-cold Buffer D supplemented with Proteinase Inhibitor Cocktail.

**Table:** Buffer D

Reagent	Final concentration	Amount
HEPES-KOH, pH 7.9 (1 M)	30 mM	1.5 mL
Potassium acetate (2.5 M)	120 mM	2.4 mL
Magnesium acetate (1 M)	3.5 mM	175 μL
DTT (1 M)	2 mM	100 μL
CHAPS (1% (w/v))	0.01% (w/v)	0.5 mL
Nuclease-free water	N/A	45.3 mL
Total	N/A	50 mL

Buffer without DTT can be stored at room temperature (20°C−25°C) up to 3 months.
  c. Add S100 to the beads and capture FLAG-AGO2 protein by rotating the beads for 2 h at 4°C.
  d. Briefly wash the beads three times with 1 mL room-temperature Buffer D supplemented with Proteinase Inhibitor Cocktail.
  e. Load immobilized AGO2 by incubating the beads with 1 μM single-stranded, 5′-phosphorylated synthetic miR-449a guide (5′-UGG CAG UGU AUU GUU AGC UGG U; resuspended in water) in 250 μL Buffer D rotating for 1 h at 37°C.
  f. Briefly wash the beads three times with 1 mL room-temperature Buffer D to remove unbound synthetic miRNA.
  g. Incubate the beads rotating for 1 h at room temperature (20°C−25°C) with 100 ng·μL^−1^ 3XFLAG peptide in 400 μL Buffer D.
  h. Collect the eluate and place it on ice.
  i. Repeat elution.
  j. Combine both eluates, comprising unloaded protein and AGO2 loaded with exogenous or endogenous small RNA guides.
4. Purify RISCs as described^8^ (Figure 1).
  ***Note:*** RISC is captured using an RNA oligonucleotide containing a high affinity binding site (complementary to miRNA nucleotides (nt) 2−8 and 13−16). RISC is eluted by adding an excess of competitor cDNA oligonucleotide, which is perfectly complementary to the capture oligonucleotide. The strategy takes advantage of the finding that dissociation of RISC from a target RNA is > 1,000 times more rapid then melting of an RNA:DNA duplex.^8^^,^^9^
  a. Use 20 μL streptavidin paramagnetic beads (Dynabeads MyOne Streptavidin T1, Life Technologies) per mL S100.
  b. Bind a biotinylated, 2′-*O*-methyl capture oligonucleotide (5′-GAU CAA CAA UAA CCC ACC ACU GCC UAU AGA; complementary to miRNA nucleotides 2−8 and 13−16) to the beads according to the manufacturer’s instructions.
  c. Incubate the assembled RISC (step 3j) with the beads rotating 1 h at room temperature (20°C−25°C) or overnight (12–18 h) at 4°C.
  d. Prepare Buffers E and F. Buffers without DTT can be stored at room temperature (20°C−25°C) up to 3 months.

**Table:** Buffer E

Reagent	Final concentration	Amount
HEPES-KOH, pH 7.9 (1 M)	30 mM	1.5 mL
Potassium acetate (2.5 M)	2 M	40 mL
Magnesium acetate (1 M)	3.5 mM	175 μL
DTT (1 M)	2 mM	100 μL
CHAPS (1% (w/v))	0.01% (w/v)	0.5 mL
Nuclease-free water	N/A	7.7 mL
Total	N/A	50 mL

**Table:** Buffer F

Reagent	Final concentration	Amount
HEPES-KOH, pH 7.9 (1 M)	30 mM	1.5 mL
Potassium acetate (2.5 M)	1 M	20 mL
Magnesium acetate (1 M)	3.5 mM	175 μL
DTT (1 M)	2 mM	100 μL
CHAPS (1% (w/v))	0.01% (w/v)	0.5 mL
Nuclease-free water	N/A	27.7 mL
Total	N/A	50 mL
  e. Briefly wash the beads five times with 1 mL Buffer D.
  f. Briefly wash the beads five times with 1 mL Buffer E.
  g. Elute RISC with 10 μM biotinylated competitor oligonucleotide (5′-TCT ATA GGC AGT GGT GGG TTA TTG TTG ATC; perfectly complementary to 2′-*O*-methyl capture oligonucleotide) in 100 μL Buffer F for 1 h at 37°C.
  h. Collect the supernatant and keep on ice.
  i. Repeat elution.
  j. Combine both eluates.
  k. Remove excess competitor oligonucleotide by rotating the eluate from step 4j with 250 μL streptavidin paramagnetic beads (Dynabeads MyOne Streptavidin T1, Life Technologies) for 15 min at room temperature (20°C−25°C).
  l. Dialyze supernatant from step 4k at 4°C against three changes (3 h each) of a 3,000-fold excess of Buffer D supplemented with 20% (w/v) glycerol.
  m. Aliquot RISC into ice-cold tubes (10 μL per aliquot).
  n. Flash-freeze in liquid nitrogen and store at −80°C.
5. Determine total concentration of RISC by quantitative northern hybridization as described.^10^^,^^11^
  a. Resolve miRNA guide standards (synthetic miR-449a at 0.5 nM, 1 nM, 2.5 nM, 5 nM, 10 nM, 25 nM, and 50 nM in Buffer D supplemented with 20% (w/v) glycerol) and RISC on a denaturing 15% polyacrylamide gel (19 cm × 16 cm × 0.4 mm).
  b. Transfer RNA to Hybond-XL (Cytiva) by semi-dry transfer at 20 V for 1 h.
  c. Crosslink RNA to the membrane with 0.16 M EDC in 0.13 M 1-methylimidazole (pH 8.0) at 60°C for 1 h.
  d. Pre-hybridize the crosslinked membrane in Church Buffer (1% (w/v) BSA, 1 mM EDTA, 0.5 M Phosphate Buffer, and 7% (w/v) SDS) at 37°C for 1 h.
  e. Add radiolabeled, 25 pmol 5′ ^32^P-DNA probe (5ʹ-ACC AGC TAA CAA TAC ACT GCC A; perfectly complementary to miR-449a) in Church Buffer to the membrane.
  f. Allow the probe to hybridize overnight (12–18 h) at 37°C.
  g. Wash the membrane twice with 2× SSC containing 0.1% (w/v) SDS and twice with 1× SSC containing 0.1% (w/v) SDS at 37°C for 15 min.
  h. Air dry the membrane and expose to a storage phosphor screen.

## Key resources table

**Table:** 

REAGENT or RESOURCE	SOURCE	IDENTIFIER
**Chemicals, peptides, and recombinant proteins**		

Ethylenediaminetetraacetic acid (EDTA)	Sigma-Aldrich	03620
Phenol:chloroform 5:1, for molecular biology	Sigma-Aldrich	P1944
N,N,N′,N′-Tetramethylethylenediamine (TEMED)	Sigma-Aldrich	T9281
Ammonium persulfate for molecular biology	Sigma-Aldrich	A3678
dNTP Set (100 mM)	Thermo Fisher Scientific	10297018
[γ-^32^P] ATP (6000 Ci·mmol^–1^, 150 mCi·ml^–1^, 5 mCi)	PerkinElmer	NEG035C005MC
SequaGel – Urea Gel Concentrate	National Diagnostics	EC-830
Adenosine 5′ triphosphate (ATP)	Sigma-Aldrich	A7699-1G
Chloroform	Thermo Fisher Scientific	AC423555000
Ethanol	Lab Supermarket	04355223EA
1-(3-Dimethylaminopropyl)-3-ethylcarbodiimide hydrochloride (EDC-HCL)	Thermo Fisher Scientific	25952-53-8
1-Methylimidazole	Sigma-Aldrich	M50834-100G
Hydrochloric acid solution	Thermo Fisher Scientific	7647-01-0
Sodium dodecyl sulfate (SDS)	Thermo Fisher Scientific	NC9983815
HEPES ultra-pure, free acid	Bio Basic	HB0264
Potassium acetate	Sigma-Aldrich	P5708-1KG
Magnesium acetate	Sigma-Aldrich	228648
Dithiothreitol (DTT)	Bio Basic	DB0058
AEBSF, hydrochloride	Sigma-Aldrich	101500
Aprotinin	Bio Basic	AD0153
Bestatin, hydrochloride	Sigma-Aldrich	B8385
E-64	VWR	97063-252
Leupeptin, hemisulfate	Sigma-Aldrich	108975
Sodium hydroxide solution (10 N)	Thermo Fisher Scientific	NC9433345
Potassium hydroxide	Sigma-Aldrich	P1767-1KG
Transfer ribonucleic acid (tRNA)	Sigma-Aldrich	R9001
RNasin Plus RNase Inhibitor, 10,000 U	Promega Corporation	N2615
UltraPure BSA (50 mg·ml^–1^)	Life Technologies	AM2618
Sodium acetate anhydrous, for molecular biology	Sigma-Aldrich	S2889-250G
Proteinase K	EMD Millipore	70663-5

**Critical commercial assays**		

MinElute Gel Extraction Kit	Qiagen	28606
NextSeq 500/550 High Output Kit v2.5 (75 Cycles)	Illumina	20024906
Agilent High Sensitivity DNA Kit	Agilent	5067-4626
Costar Spin-X columns	Thermo Fisher Scientific	07200386
T4 Polynucleotide Kinase, 2,500 U	New England Biolabs	M0201L
Microspin G-25 columns	VWR	95017-621
Dynabeads MyOneStreptavidin T1	Life Technologies	65602
Mini Dialysis Kit, mini 12,000	Sigma-Aldrich	PURN12100-1KT
Hybond-XL membrane 30 cm × 3 m roll	Thermo Fisher Scientific	45001148
Protran nitrocellulose	Millipore Sigma	GE10600002
20× SSC Liquid Concentrate	Bio Basic	D0623
7X-O-Matic Cleaning Solution	VWR	76-674-94
PCR 8-Tube Strips	USA Scientific	1402-4700
SuperScript III Reverse Transcriptase	Life Technologies	18080085
AccuPrime Pfx DNA Polymerase	Life Technologies	12344024
Certified Low Range Ultra Agarose	Bio-Rad Laboratories	161-3107
Cell scrapers	Thermo Fisher Scientific	NC1890484
MicroSpin G-25 Columns	Cytiva	27532501
Chromatography paper, thick	Fisher Scientific	05-714-4

**Experimental models: Cell lines**		

HEK 293T stably overexpressing FLAG-tagged mouse AGO2	Jouravleva et al.^1^	N/A

**Oligonucleotides**		

Table 1	This study	N/A

**Software and algorithms**		

Estimation of *K*_D_ values	Jouravleva et al.^1^	https://doi.org/10.6084/m9.figshare.19180952

**Other**		

ORBI-Shaking	Benchmark	BT1011
Cooling microcentrifuge	Eppendorf	5417R
Spectrophotometer	Beckman Coulter	DU 640B
DynaMag-2 Magnet	Thermo Fisher Scientific	12321D
Thermocycler with a heated lid	Bio-Rad	C1000 Touch
Agilent 2100 Bioanalyzer High Sensitivity	Agilent Technologies	G2939A
NextSeq 500	Illumina	N/A
Electrophoresis power supply	Thermo Electron Corporation	EC3000P
Bio-Dot microfiltration apparatus	Bio-Rad	170-6545
Typhoon FLA 7000	GE HealthCare	N/A

**Table 1:** RNA and DNA Oligos used in this study

RISC loading & purification	Sequence (m, 2′-*O*-methyl; p, 5′ phosphate; Bio, biotin-6-carbon spacer)
Guide strand for miR-449a RISC	pUGG CAG UGU AUU GUU AGC UGG U
Capture Oligo to affinity purify miR-449a RISC	Bio-mGmAmU mCmAmA mCmAmA mUmAmA mCmCmC mAmCmC mAmCmU mGmCmC mUmAmU mAmGmA
DNA competitor to elute miR-449a RISC	Bio-TTA TAG GCA GTG GTG GGT TAT TGT TGA TC

RISC quantification	Sequence (p, 5′ phosphate)
DNA probe to quantify total concentration of miR-449a RISC by Northern Blot	ATG ACC AGC TAA CAA TAC ACT GCC AAC T
RNA target to quantify active concentration of miR-449a RISCs by double-filter binding	pAUG AAA UCG AUA UCU AUC ACU GCC AAC U

RBNS	Sequence (p, 5′ phosphate)
RBNS RNA input pool	pGAG UUC UAC AGU CCG ACG AUC NNN NNN NNN NNN NNN NNN NNU GGA AUU CUC GGG UGC CAA
5′-end blocking cDNA oligonucleotide	GTC GGA CTG TAG AAC TC
3′-end blocking cDNA oligonucleotide	TTG GCA CCC GAG AAT
RT primer	CCT TGG CAC CCG AGA ATT CCA
PCR Forward primer	AAT GAT ACG GCG ACC ACC GAG ATC TAC ACG TTC AGA GTT CTA CAG TCC GA
Multiplexing PCR Reverse Primer PCRId1	CAA GCA GAA GAC GGC ATA CGA GAT CGT GAT GTG ACT GGA GTT CCT TGG CAC CCG AGA ATT CCA
Multiplexing PCR Reverse Primer PCRId2	CAA GCA GAA GAC GGC ATA CGA GAT ACA TCG GTG ACT GGA GTT CCT TGG CAC CCG AGA ATT CCA
Multiplexing PCR Reverse Primer PCRId3	CAA GCA GAA GAC GGC ATA CGA GAT GCC TAA GTG ACT GGA GTT CCT TGG CAC CCG AGA ATT CCA
Multiplexing PCR Reverse Primer PCRId4	CAA GCA GAA GAC GGC ATA CGA GAT TGG TCA GTG ACT GGA GTT CCT TGG CAC CCG AGA ATT CCA
Multiplexing PCR Reverse Primer PCRId5	CAA GCA GAA GAC GGC ATA CGA GAT CAC TGT GTG ACT GGA GTT CCT TGG CAC CCG AGA ATT CCA
Multiplexing PCR Reverse Primer PCRId6	CAA GCA GAA GAC GGC ATA CGA GAT ATT GGC GTG ACT GGA GTT CCT TGG CAC CCG AGA ATT CCA
Multiplexing PCR Reverse Primer PCRId7	CAA GCA GAA GAC GGC ATA CGA GAT GAT CTG GTG ACT GGA GTT CCT TGG CAC CCG AGA ATT CCA
Multiplexing PCR Reverse Primer PCRId8	CAA GCA GAA GAC GGC ATA CGA GAT TCA AGT GTG ACT GGA GTT CCT TGG CAC CCG AGA ATT CCA
Multiplexing PCR Reverse Primer PCRId9	CAA GCA GAA GAC GGC ATA CGA GAT CTG ATC GTG ACT GGA GTT CCT TGG CAC CCG AGA ATT CCA
Multiplexing PCR Reverse Primer PCRId10	CAA GCA GAA GAC GGC ATA CGA GAT AAG CTA GTG ACT GGA GTT CCT TGG CAC CCG AGA ATT CCA
Multiplexing PCR Reverse Primer PCRId11	CAA GCA GAA GAC GGC ATA CGA GAT GTA GCC GTG ACT GGA GTT CCT TGG CAC CCG AGA ATT CCA
Multiplexing PCR Reverse Primer PCRId12	CAA GCA GAA GAC GGC ATA CGA GAT TAC AAG GTG ACT GGA GTT CCT TGG CAC CCG AGA ATT CCA
Multiplexing PCR Reverse Primer PCRId13	CAA GCA GAA GAC GGC ATA CGA GAT TTG ACT GTG ACT GGA GTT CCT TGG CAC CCG AGA ATT CCA
Multiplexing PCR Reverse Primer PCRId14	CAA GCA GAA GAC GGC ATA CGA GAT GGA ACT GTG ACT GGA GTT CCT TGG CAC CCG AGA ATT CCA
Multiplexing PCR Reverse Primer PCRId15	CAA GCA GAA GAC GGC ATA CGA GAT TGA CAT GTG ACT GGA GTT CCT TGG CAC CCG AGA ATT CCA
Multiplexing PCR Reverse Primer PCRId16	CAA GCA GAA GAC GGC ATA CGA GAT GGA CGG GTG ACT GGA GTT CCT TGG CAC CCG AGA ATT CCA
Multiplexing PCR Reverse Primer PCRId17	CAA GCA GAA GAC GGC ATA CGA GAT CTC TAC GTG ACT GGA GTT CCT TGG CAC CCG AGA ATT CCA
Multiplexing PCR Reverse Primer PCRId18	CAA GCA GAA GAC GGC ATA CGA GAT GCG GAC GTG ACT GGA GTT CCT TGG CAC CCG AGA ATT CCA
Multiplexing PCR Reverse Primer PCRId19	CAA GCA GAA GAC GGC ATA CGA GAT TTT CAC GTG ACT GGA GTT CCT TGG CAC CCG AGA ATT CCA
Multiplexing PCR Reverse Primer PCRId20	CAA GCA GAA GAC GGC ATA CGA GAT GGC CAC GTG ACT GGA GTT CCT TGG CAC CCG AGA ATT CCA
Multiplexing PCR Reverse Primer PCRId21	CAA GCA GAA GAC GGC ATA CGA GAT CGA AAC GTG ACT GGA GTT CCT TGG CAC CCG AGA ATT CCA
Multiplexing PCR Reverse Primer PCRId22	CAA GCA GAA GAC GGC ATA CGA GAT CGT ACG GTG ACT GGA GTT CCT TGG CAC CCG AGA ATT CCA
Multiplexing PCR Reverse Primer PCRId23	CAA GCA GAA GAC GGC ATA CGA GAT CCA CTC GTG ACT GGA GTT CCT TGG CAC CCG AGA ATT CCA
Multiplexing PCR Reverse Primer PCRId24	CAA GCA GAA GAC GGC ATA CGA GAT GCT ACC GTG ACT GGA GTT CCT TGG CAC CCG AGA ATT CCA
Multiplexing PCR Reverse Primer PCRId25	CAA GCA GAA GAC GGC ATA CGA GAT ATC AGT GTG ACT GGA GTT CCT TGG CAC CCG AGA ATT CCA
Multiplexing PCR Reverse Primer PCRId26	CAA GCA GAA GAC GGC ATA CGA GAT GCT CAT GTG ACT GGA GTT CCT TGG CAC CCG AGA ATT CCA
Multiplexing PCR Reverse Primer PCRId27	CAA GCA GAA GAC GGC ATA CGA GAT AGG AAT GTG ACT GGA GTT CCT TGG CAC CCG AGA ATT CCA
Multiplexing PCR Reverse Primer PCRId28	CAA GCA GAA GAC GGC ATA CGA GAT CTT TTG GTG ACT GGA GTT CCT TGG CAC CCG AGA ATT CCA
Multiplexing PCR Reverse Primer PCRId29	CAA GCA GAA GAC GGC ATA CGA GAT TAG TTG GTG ACT GGA GTT CCT TGG CAC CCG AGA ATT CCA
Multiplexing PCR Reverse Primer PCRId30	CAA GCA GAA GAC GGC ATA CGA GAT CCG GTG GTG ACT GGA GTT CCT TGG CAC CCG AGA ATT CCA
Multiplexing PCR Reverse Primer PCRId31	CAA GCA GAA GAC GGC ATA CGA GAT ATC GTG GTG ACT GGA GTT CCT TGG CAC CCG AGA ATT CCA
Multiplexing PCR Reverse Primer PCRId32	CAA GCA GAA GAC GGC ATA CGA GAT TGA GTG GTG ACT GGA GTT CCT TGG CAC CCG AGA ATT CCA
Multiplexing PCR Reverse Primer PCRId33	CAA GCA GAA GAC GGC ATA CGA GAT CGC CTG GTG ACT GGA GTT CCT TGG CAC CCG AGA ATT CCA
Multiplexing PCR Reverse Primer PCRId34	CAA GCA GAA GAC GGC ATA CGA GAT GCC ATG GTG ACT GGA GTT CCT TGG CAC CCG AGA ATT CCA
Multiplexing PCR Reverse Primer PCRId35	CAA GCA GAA GAC GGC ATA CGA GAT AAA ATG GTG ACT GGA GTT CCT TGG CAC CCG AGA ATT CCA
Multiplexing PCR Reverse Primer PCRId36	CAA GCA GAA GAC GGC ATA CGA GAT TGT TGG GTG ACT GGA GTT CCT TGG CAC CCG AGA ATT CCA

## Materials and equipment

**Table:** Storage Buffer

Reagent	Final concentration	Amount
HEPES-KOH, pH 7.9 (1 M)	30 mM	30 μL
Potassium acetate (2.5 M)	120 mM	48 μL
Magnesium acetate (1 M)	3.5 mM	3.5 μL
DTT (1 M)	2 mM	2 μL
CHAPS (1% (w/v))	0.01% (w/v)	10 μL
Glycerol (50% (w/v))	20% (w/v)	400 μL
Nuclease-free water	N/A	506.5 μL
Total	N/A	1,000 μL

Buffer without DTT can be stored at room temperature (20°C−25°C) up to 3 months.

**Table:** Dilution Buffer

Reagent	Final concentration	Amount
HEPES-KOH, pH 7.9 (1 M)	30 mM	30 μL
Potassium acetate (2.5 M)	120 mM	48 μL
Magnesium acetate (1 M)	3.5 mM	3.5 μL
DTT (1 M)	1 mM	1 μL
CHAPS (1% (w/v))	0.01% (w/v)	10 μL
tRNA (2 mg·mL^−1^)	0.01 mg·mL^−1^	5 μL
Nuclease-free water	N/A	902.5 μL
Total	N/A	1,000 μL

Prepare immediately before use and store it at 4°C.

**Table:** Reaction Mix Buffer (10×)

Reagent	Final concentration	Amount
HEPES-KOH, pH 7.9 (1 M)	216 mM	216 μL
Potassium acetate (2.5 M)	1033 mM	413.2 μL
DTT (1 M)	20 mM	20 μL
Nuclease-free water	N/A	350.8 μL
Total	N/A	1,000 μL

Prepare immediately before use and store it at 4°C.

**Table:** Wash Buffer

Reagent	Final concentration	Amount
HEPES-KOH, pH 7.9 (1 M)	30 mM	3 mL
Potassium acetate (2.5 M)	120 mM	4.8 mL
Magnesium acetate (1 M)	3.5 mM	350 μL
DTT (1 M)	1 mM	100 μL
Nuclease-free water	N/A	to 100 mL
Total	N/A	100 mL

Buffer without DTT can be stored at room temperature (20°C−25°C) up to 3 months.

**Table:** Proteinase K Buffer (2×)

Reagent	Final concentration	Amount
Tris-HCl (1 M)	200 mM	50 mL
EDTA, pH 8.0 (0.5 M)	20 mM	10 mL
NaCl (5 M)	300 mM	15 mL
SDS	2% (w/v)	5 g
Nuclease-free water	N/A	to 250 mL
Total	N/A	250 mL

Buffer can be stored at room temperature (20°C−25°C) up to 3 months.

## Step-by-step method details

### General guidelines for preparing target RNA

**Timing: 2−36 h**

The procedure below describes preparation of 5′-end labeled target RNA in two steps: (1) dephosphorylation of 5′ phosphorylated synthetic RNA oligonucleotides followed by (2) 5′-end labeling with radioactive ATP ([γ-^32^P]ATP) using T4 Polynucleotide Kinase (PNK).

***Note:*** To achieve high sensitivity, we radioactively 5′-end label the target RNA. A typical double filter-binding assay or RBNS experiment requires ∼30 fmol and ∼20 pmol of target RNA, respectively. To ensure a high concentration of target RNA, we radioactively label a small amount (e.g., 100 pmol) of target RNA and mix it with cold target RNA to reach the amount required in an assay. If the experimentalist purchases target RNA both with 5′ phosphate and without, proceed directly with 5′-end labeling (step 2). For best results, do not use a radioactive batch of [γ-^32^P]ATP that is more than 3 weeks old.

**CRITICAL:** DEPC-treated water should not be used, because it can be acidic and may cause depurination. Unreacted DEPC or by-products from autoclaving can also inhibit enzymes.

1. Dephosphorylate 5′ phosphorylated synthetic RNA oligonucleotides.
  a. Set up the reaction by mixing:

**Table:** 

Antarctic Phosphatase Buffer (10×)	2.5 μL
5′ phosphorylated RNA oligonucleotide (100 μM)	1 μL
Antarctic Phosphatase (5 U·μL^−1^)	2 μL
Nuclease-free water	19.5 μL
**Total**	25 μL

    ***Note:*** The table shows the volumes to dephosphorylate 100 pmol RNA. Linearly scale-up accordingly to amount needed.
  b. Incubate at 37°C for 30 min.
  c. Inactivate the Antarctic Phosphatase by adding 50 mM EDTA (f.c.) and heating at 80°C for 2 min. Cool at room temperature (20°C−25°C) for 1 min.
  d. Add RNase-free water to adjust sample volume to 300 μL.
  e. Precipitate the RNA by adding 0.1 volumes of 3 M sodium acetate (pH 5.2) and three volumes of ethanol.
  f. Incubate the solution 2 h at −80°C or overnight (12–18 h) at −20°C.
  g. Spin at the maximum speed (typically 21,000 × *g*) for 30 min at 4°C in a microcentrifuge.
  h. Carefully remove nearly all supernatant.
  i. Add 1 mL of ice-cold 75% ethanol to RNA. Mix well by vigorous vortexing.
  j. Spin at the maximum speed for 15 min at 4°C in a microcentrifuge.
  k. Carefully remove nearly all supernatant.
  l. Repeat steps 1i–k.
  m. Spin at the maximum speed for 15 s at 4°C in a microcentrifuge to collect the residual ethanol on the wall of the tube and discard the liquid using an extended pipette tip.
  n. Air-dry the pellet for 5−20 min.
  o. Resuspend the pellet in 10 μL nuclease-free water.
2. Set up a 5′-end labeling reaction.
  a. To dephosphorylated RNA oligonucleotide (from step 1o), add:
    i. 20 μL nuclease-free water,
    ii. 4 μL 10× PNK Reaction Buffer,
    iii. 2 μL 10 U·μL^−1^ T4 PNK,
    iv. 4 μL [γ-^32^P]ATP (25 pmol·μL^−1^).
      **CRITICAL:** The protocol involves the use of radiolabeled nucleotides; follow institutional guidelines for working with radionuclides.
  b. Incubate at 37°C for 30 min.
    ***Note:*** To ensure that all target RNA molecules are 5′-phosphorylated, follow the radiolabeling with a second phosphorylation reaction containing 2 mM non-radioactive ATP. To the radiolabeling reaction, add:
    i. 7 μL nuclease-free water,
    ii. 1 μL 10× PNK Reaction Buffer,
    iii. 1 μL 100 mM ATP,
    iv. 1 μL 10 U·μL^−1^ T4 PNK.
      Incubate at 37°C for 30 min.
3. Remove unincorporated [γ-^32^P]ATP with a Sephadex G-25 spin column according to the manufacturer’s instructions. Save the empty tube of labeling reaction for preparation of radioactive markers (optional step below).
  ***Optional:*** we highly recommend gel purifying target RNAs to remove traces of unincorporated [γ-^32^P]ATP and degraded RNA.
  a. Resolve 28-nt target RNA and RNA for RBNS on 10% and 8% polyacrylamide urea gels, respectively. For more details on how to prepare, load, and run polyacrylamide gels for RNA analysis, we refer the experimentalist to existing protocols.^12^
  b. Visualize the radiolabeled target by phosphorimaging:
    i. Remove one glass plate and cover the gel with plastic wrap.
    ii. Prepare radioactive markers: cut three small triangular shapes out of filter paper (Figure 2).
    iii. Place radioactive markers on top of the gel.
    iv. Add ∼300 μL of Loading Buffer to the empty tube of labeling reaction saved in step 3.
    v. Add this radioactive solution dropwise to the triangular shapes; it should be evenly distributed on the triangular pieces of the filter paper.
    vi. Cover the gel and the radioactive markers with plastic wrap.
    vii. Expose the gel to a storage phosphor imaging screen for ∼15−30 s and print an image at actual size.
  c. Identify the location of the radiolabeled RNA on the gel, using the radioactive markers on the gel to line up the gel with the marks on the printed image.
  d. Excise the pieces of the gel containing the RNA with a clean razor blade.
  e. Recover RNA from the gel by the Crush and Soak method.^13^ We typically elute RNA in 300 mM NaCl, 1 mM EDTA (pH 8.2) and 1% (w/v) SDS.
4. Purify RNA by phenol/chloroform extraction followed by ethanol precipitation.
  a. If the optional step above has been performed, pass eluate through a small 0.45-μm filter into a new tube. Alternatively, load the eluate on a Spin-X filter, centrifuge at 700 × *g* for 10 min at room temperature (20°C−25°C); collect the flow-through.
  b. Add an equal volume of phenol:chloroform 5:1 (pH 4.5) to aqueous RNA solution.
    **CRITICAL:** Phenol and chloroform are toxic upon inhalation. Always handle in a chemical fume hood. Avoid contact with eyes and skin. Strictly follow institutional guidelines for waste disposal.
  c. Mix well by vigorous vortexing, and spin at the maximum speed for 15 min at room temperature (20°C−25°C) in a microcentrifuge.
  d. Transfer the aqueous (top) phase to a new 2-mL tube.
    ***Note:*** Avoid transferring any of the interphase or organic layer into the pipette when removing the aqueous phase. Angle the tube at 45°C to facilitate the process.
  e. Add an equal volume of chloroform and mix well by vigorous vortexing.
  f. Spin at the maximum speed for 15 min at room temperature (20°C−25°C) in a microcentrifuge.
  g. Transfer the aqueous (top) phase to a new 2-mL tube.
  h. Follow instructions from steps 1d−n to recover RNA by ethanol precipitation.
    ***Note:*** If RNA was gel-purified and eluted in in 300 mM NaCl, 1 mM EDTA (pH 8.2) and 1% (w/v) SDS in step 3e, there is no need to add 0.1 volumes 3 M sodium acetate (step 1e).
  i. Resuspend the RNA pellet in 40 μL of nuclease-free water.
  j. Measure absorbance at 260 nm in a spectrophotometer.
  k. Determine RNA concentration of RNA using the Beer-Lambert law:
    A=ε×l×c
    where A is absorbance at 260 nm, l is the optical path length in cm, ε is the molar extinction coefficient of RNA oligonucleotide in L·mol^−1^·cm^−1^, and c is RNA concentration in M.
    **CRITICAL:** For accurate quantification, absorbance values must be between 0.1 and 0.8. Solutions of higher and lower concentrations have higher relative error in the measurement. If absorbance is < 0.1, add cold RNA to obtain an absorbance measurement comprised between 0.1 and 0.8. If too much cold RNA has been added and measured absorbance is > 0.8, take an aliquot of RNA solution and measure absorbance of a diluted sample.
  l. Aliquot target RNA and store at −20°C up to 6 months (or until the specific radioactivity becomes low) by strictly following institutional guidelines for storage of radioactive material.

**Figure 2:**
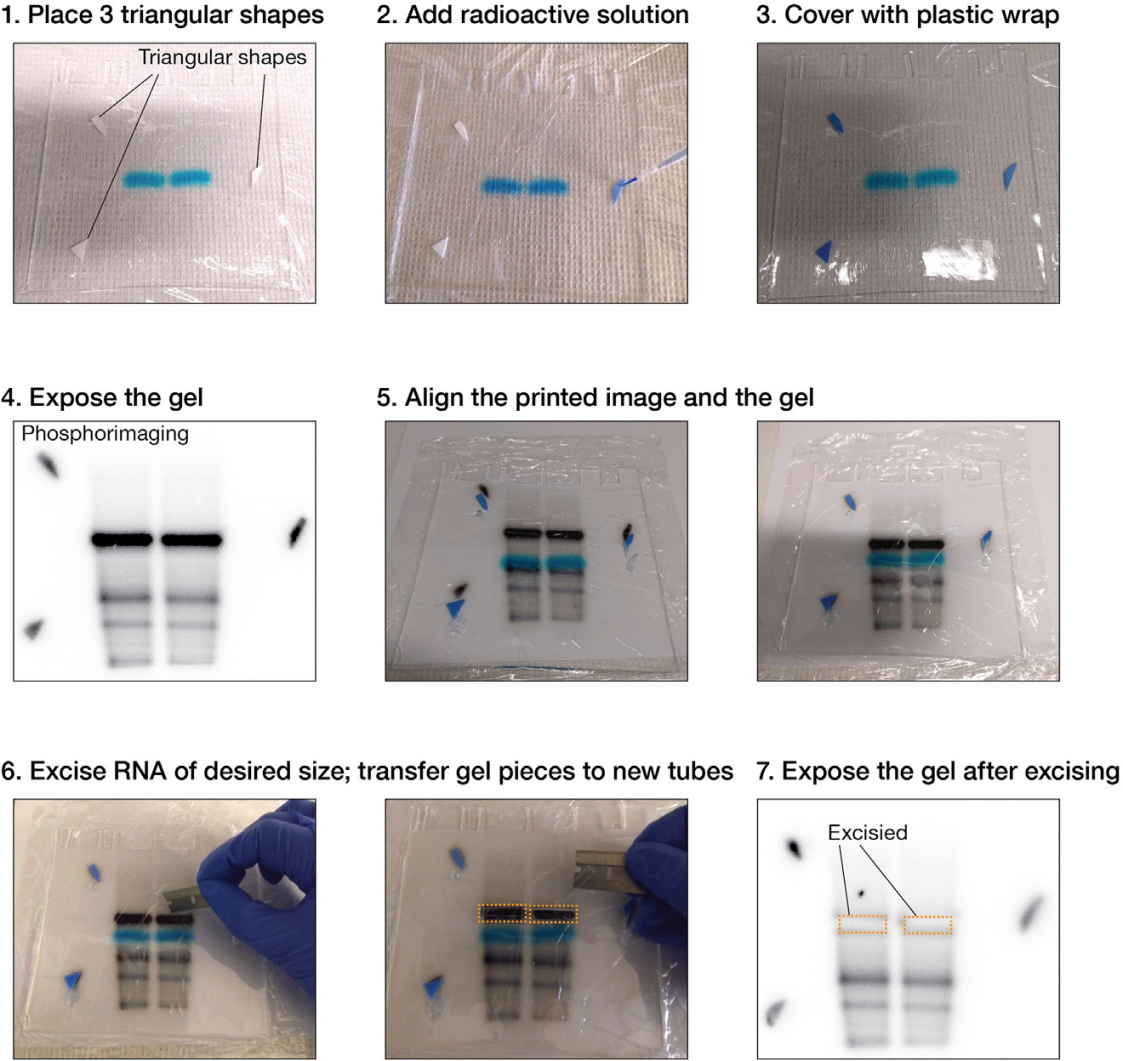
Step-by-step procedure to visualize and excise in-gel radiolabeled target by phosphorimaging

### General guidelines for handling Bio-Dot microfiltration apparatus

**Timing: 2−22.5 h**

**Figure 3:**
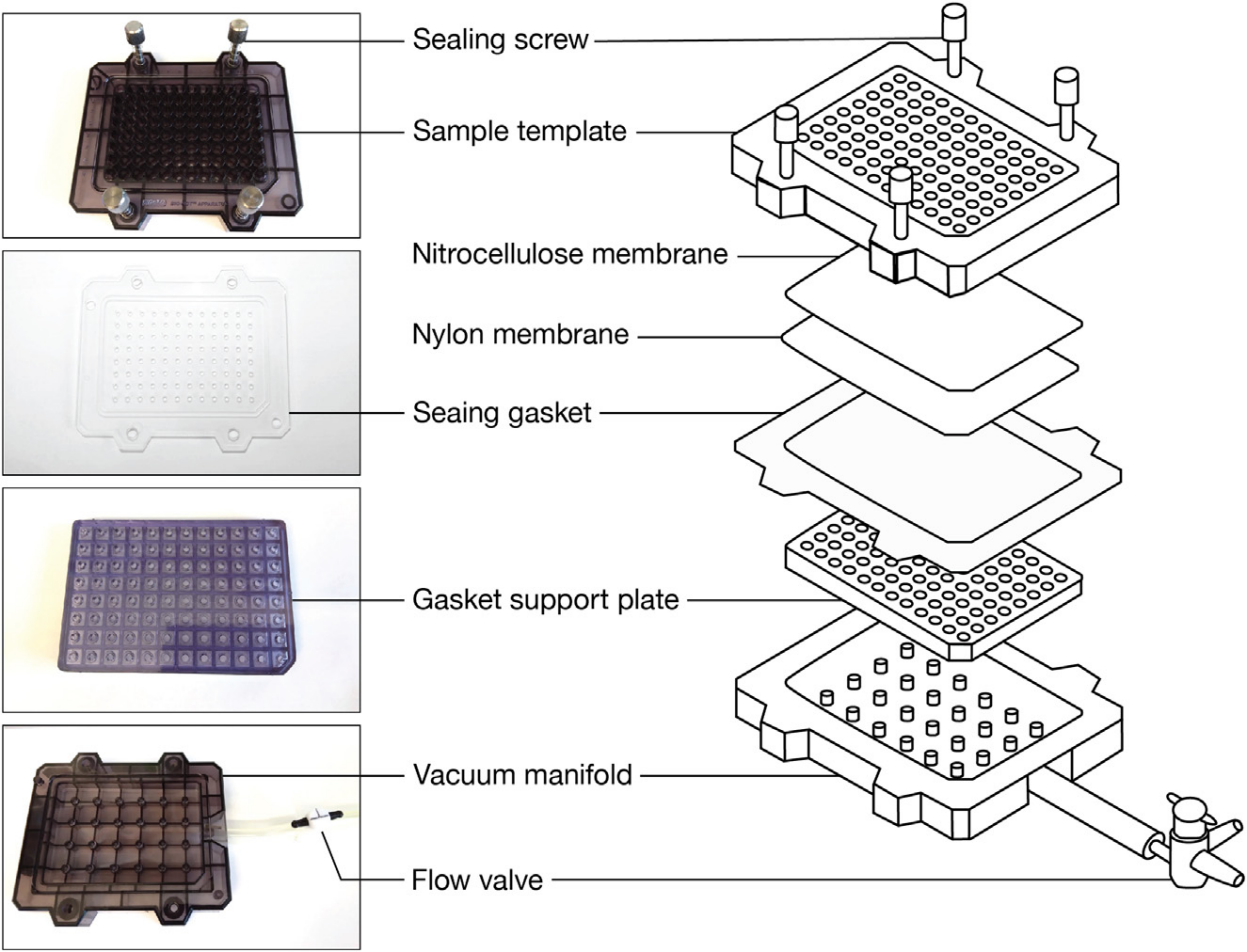
Experimental setup for double filter-binding assays using the Bio-Dot Microfiltration apparatus

Bio-Dot microfiltration apparatus is simple to operate and enables rapid separation of protein-bound RNA from unbound RNA. We describe below the handling of Bio-Dot apparatus that we use in double filter-binding assays and RBNS (Figure 3).

***Note:*** Additional information about the apparatus can be found in the manufacturer’s manual.

5. Cleaning.
  a. Soak the sample template of Bio-Dot apparatus in 0.5 M sodium hydroxide overnight (12–18 h) at room temperature (20°C−25°C) or for 1 h at 65°C to eliminate RNA and protein molecules remaining from previous assays.
  b. Wash thoroughly with RNase-free water.
  c. Wash the apparatus with a surfactant; we typically soak the apparatus in 7X-O-Matic Cleaning Solution for 15 min.
  d. Wash thoroughly with water and rinse with nuclease-free water.
  e. Air-dry the apparatus.

**Figure 4:**
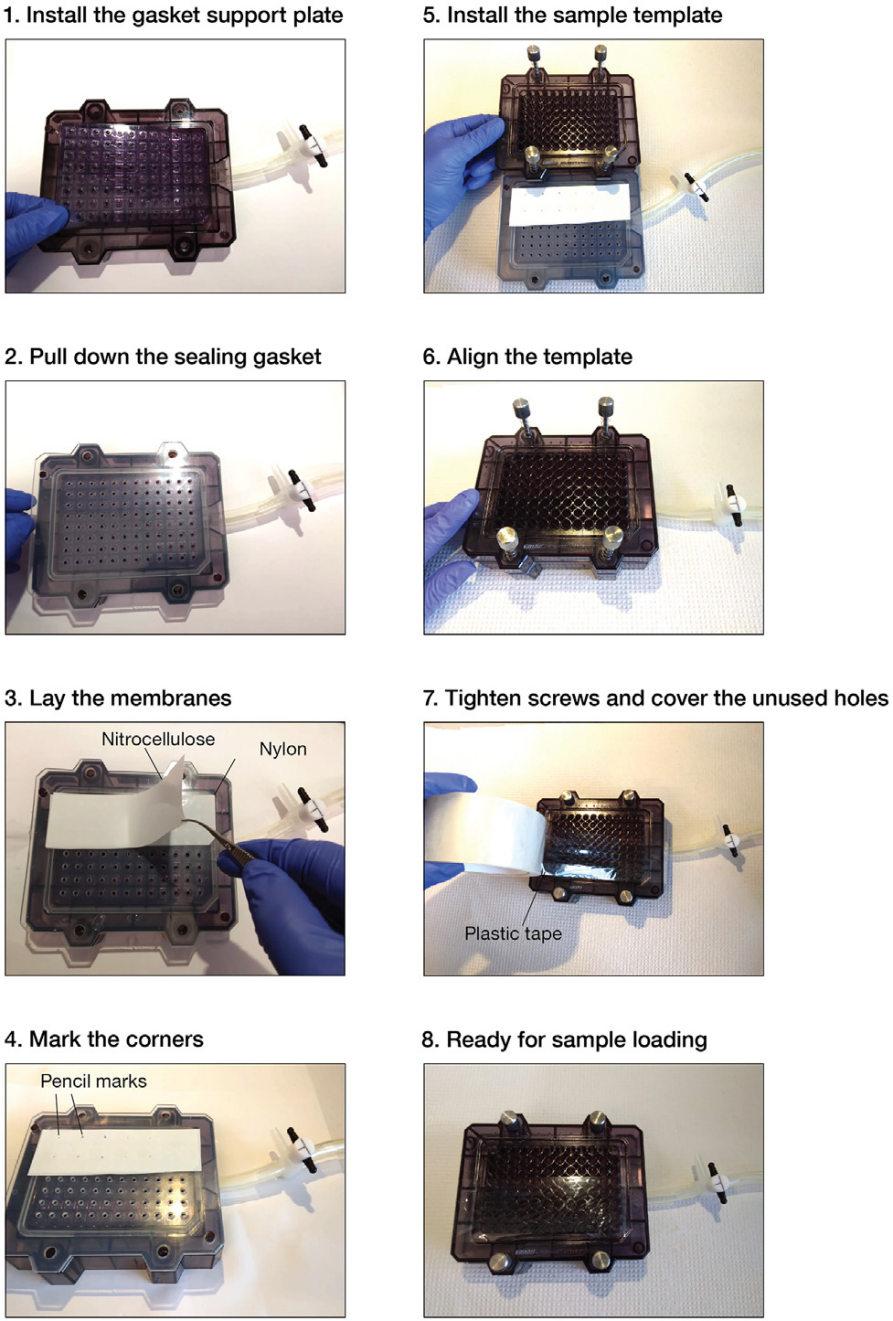
Step-by-step procedure to assemble the Bio-Dot Microfiltration apparatus for double filter-binding assays

**Figure 5:**
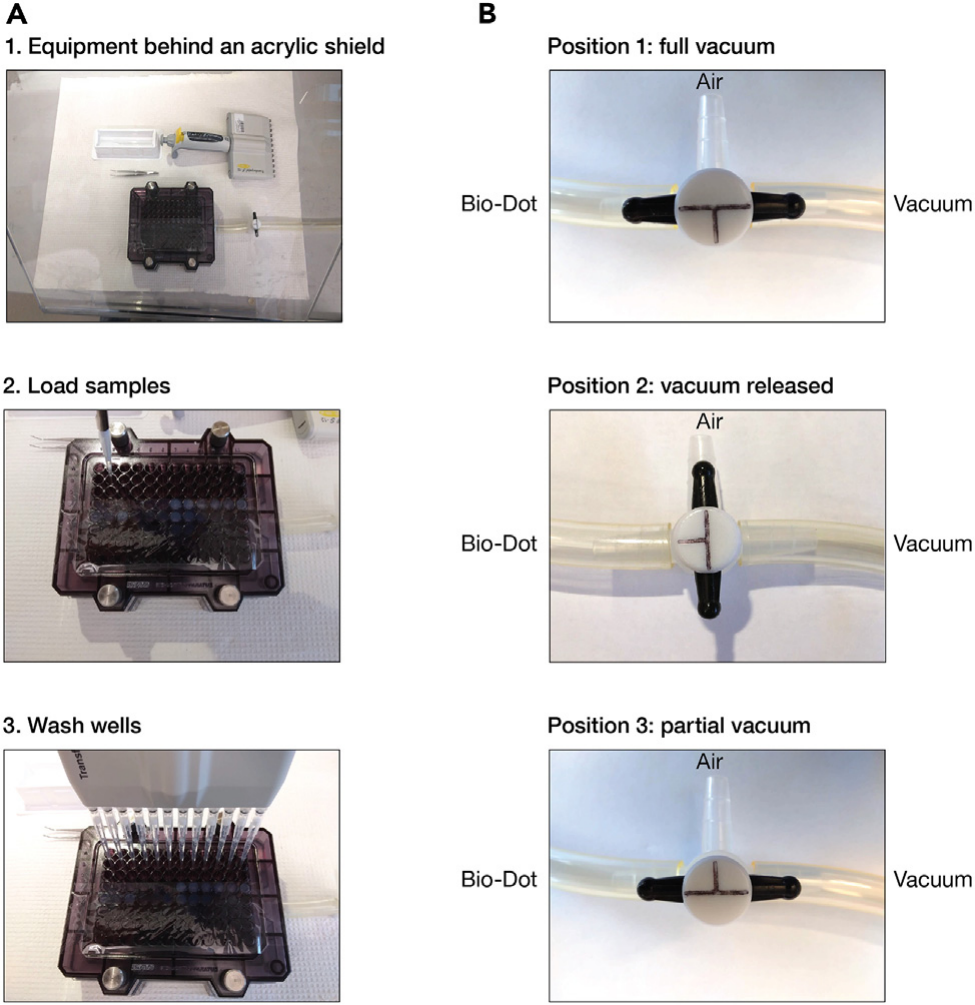
Separating protein-bound from unbound RNA (A) Illustration of fully assembled Bio-Dot Microfiltration apparatus used to apply samples followed by a rapid wash. (B) Settings for the 3-way flow valve for assembling the Bio-Dot Microfiltration apparatus, loading the samples, and washing.

**Figure 6:**
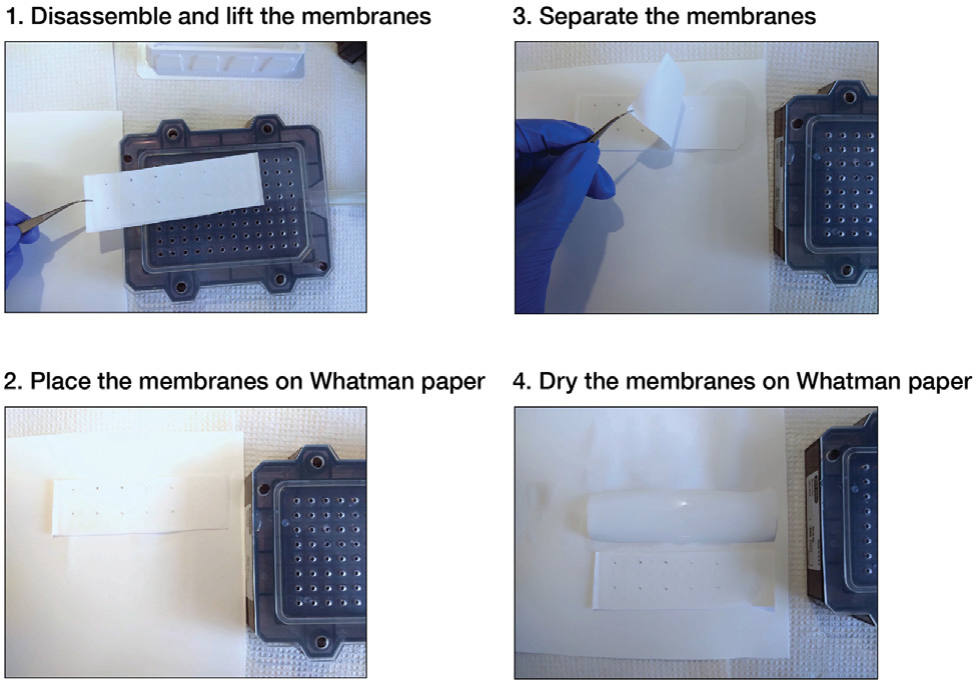
Step-by-step procedure to disassemble the Bio-Dot Microfiltration apparatus
6. Assembly.
  a. Cut each of the required membranes to fit the Bio-Dot apparatus.
    **CRITICAL:** Both nylon and nitrocellulose membranes should be the same size and should not extend beyond the edge of the gasket. Membranes of a larger size may obstruct vacuum formation after the Bio-Dot apparatus has been assembled. Trim the membranes if needed.
    ***Note:*** To reduce retention of free single-stranded RNA, pre-condition nitrocellulose membranes prior to use by soaking in 0.4 M potassium hydroxide for 10 min and washing thoroughly with nuclease-free water.^5^^,^^14^ Pre-condition nylon membranes by incubating in 0.1 M EDTA (pH 8.2) for 10 min, washing three times in 1 M sodium chloride for 10 min, rapidly rinsing (∼15 s) in 0.5 M sodium hydroxide, and washing thoroughly with nuclease-free water.^5^ We highly recommend pre-conditioning both nitrocellulose and nylon membranes in RBNS assays.
  b. Equilibrate the nitrocellulose and nylon membranes by soaking them in Wash Buffer for at least 1 h. Always wear gloves and use forceps when handing membranes.
    ***Note:*** We assess binding of mammalian RISC by performing binding reactions at 37°C. To ensure constant temperature throughout the procedure, all incubations, assembly of Bio-Dot apparatus and filtering steps are performed in a 37°C constant-temperature room, using supplies that had been pre-equilibrated to 37°C.
  c. Deposit the gasket support plate into the vacuum manifold (Figure 4).
  d. Pull down the sealing gasket on top of the vacuum manifold.
  e. Align the 96 holes in the gasket over the 96 holes in the support plate (Figure 4).
  f. Remove the nylon membrane from the Wash Buffer and shake it out gently for a few seconds to remove the excess of liquid.
    **CRITICAL:** dehydrated membranes may cause protein denaturation upon binding and may restrain proper drainage of solutions upon filtering. Therefore, remove the membrane from the wetting solution right before (≤ 5 min) filtering.
  g. Lay the nylon membrane on top of the sealing gasket (Figure 4).
  h. Remove the nitrocellulose membrane from the Wash Buffer and shake it out gently for a few seconds to remove the excess of liquid.
  i. Lay the nitrocellulose membrane on top of the nylon membrane (Figure 4).
    ***Note:*** In RBNS experiments, the nitrocellulose membrane is cut into pieces to enable extracting protein-bound RNA from each binding reaction independently. Use a pencil to draw markers around wells where samples will be applied (Figure 4). Do not accidentally drill the membrane: small holes in the membrane may impair proper vacuum formation in downstream steps.
  j. Carefully roll out any air bubbles confined between the membranes and the gasket with forceps.
  k. Place the sample template on top of the membrane (Figure 4).
  l. Finger-tighten the four screws. Use a diagonal crossing pattern to apply uniform pressure on the membrane surface.
    **CRITICAL:** if some wells are not used, they must be closed off to ensure proper vacuum to the wells in use. We cover the unused portion of the apparatus with clear packing tape to prevent air from moving through those wells (Figures 4 and 5A).
  m. Switch the vacuum source on and adjust air aspiration to gentle flow.
    ***Note:*** We are using an in-house vacuum line; alternatively, an electronic laboratory vacuum pump can be used.
  n. Adjust the flow valve so that the manifold is exposed to air only (Figure 5B; the flow valve in position 2).
  o. Connect the apparatus to the vacuum source.
  p. Place a multi-channel pipet (10−100 μL) and ice-cold Wash Buffer near the Bio-Dot apparatus (Figure 5A).
  q. When ready for sample application, remove the excess of liquid from membranes by applying gentle vacuum to the manifold (Figure 5B; the flow valve in position 3).
    ***Note:*** Loose tightening of the apparatus results in leaking between wells and cross contamination with samples. If needed, adjust screw tightening using the diagonal crossing pattern while gentle vacuum is on.
    **CRITICAL:** perform the above step rapidly so that not to dehydrate the membranes. Watch the sample wells. If membranes have dried, rehydrate the wells with some Wash Buffer (∼100 μL) and remove the excess of liquid by applying gentle vacuum to the manifold (the flow valve in position 3).
  r. Watch the sample wells. As soon as the buffer solution drains from all the wells, set up the flow valve so that the manifold is exposed to air (Figure 5B; the flow valve in position 2). Imminently proceed to the next step.
7. Loading samples.
  a. Rapidly load the samples into the wells with a multi-channel pipet.
    ***Note:*** If binding reactions are performed in a small volume < 50 μL, apply the sample in the center of the well (and not on the well walls). Ensure that there are no air bubbles in the wells, as they will prevent the sample from binding to the membrane. Air bubbles may be removed by pipetting the liquid in the well up and down.
    **CRITICAL:** RBNS is a highly sensitive assay; therefore, skip a well between samples to prevent cross-contamination.
  b. Set up the flow valve so that the manifold is exposed to the vacuum (Figure 5B; the flow valve in position 1).
  c. Watch the sample wells. After the samples drain from all the wells, leave the flow valve in position 1 and rapidly add 10−100 μL of ice-cold Wash Buffer into the wells.
  d. Watch the sample wells. When the Wash Buffer drains from all the wells and the membranes are dehydrated, the manifold is ready for disassembly.
8. Disassembly.
  a. Place a sheet of filter paper near the Bio-Dot apparatus.
  b. While the manifold is exposed to the vacuum (the flow valve in position 1), finger-loosen the four screws using a diagonal crossing pattern.
  c. Lift the sample template and turn the vacuum off by setting up the flow valve to position 2.
  d. Grip both nitrocellulose and nylon membranes together by a corner with forceps and pull them horizontally towards to the filter paper (Figure 6). The RNA side should face up.
  e. Carefully separate the membranes by lifting the nitrocellulose membrane and placing it RNA face up on the filter paper (Figure 6).
    ***Note:*** The filter paper drains excess of Wash Buffer from the membranes.
  f. Carefully transfer the membranes to a new piece of filter paper (Figure 6).
    **CRITICAL:** Air-dry the membranes (∼30 min). If wet membranes are used in the next step, the sample dots may expand by diffusion yielding halos around the samples and an eventual cross-contamination.
  g. Place a plastic wrap over the membranes.
  h. Expose the membranes to a storage phosphor imaging screen for 10 s to 15 min and for 2−16 h in RBNS and double filter-binding assays, respectively.
    ***Note:*** The signals must be distinguishable over the background, but not saturated.

### Measuring the fraction of active RISC by titration

**Timing: 5−6 days**

We describe below a titration experiment to quantify the concentration of binding-competent protein, which is a key step for determining the protein concentrations to use in double filter-binding and RBNS reactions.

***Note:*** The amount of bound ligand in double filter-binding assays and RBNS experiments is determined not by the total protein concentration but by the concentration of total active protein. Different protein preparations can have different percent activities for the same amount of protein due to misfolding, aggregation, degradation, and potentially inactivation upon purification. We estimate total RISC concentration by northern blot and measure the equilibrium binding of active RISC with a high-affinity RNA target. Concentration of RNA target is chosen to be much greater than its known *K*_D_, and RISC concentration is varied by an order of magnitude above and below the target concentration. Fitting the titration data to a quadratic equation enables measuring the amount of active RISC.

9. Design a target RNA with a sequence complementary to a high-affinity binding site; we use a target RNA with an 8mer binding site (a target RNA with Watson-Crick pairing to miRNA nucleotides at positions 2−8 and an adenosine opposite miRNA nucleotide at position 1).
10. Add flanking regions (≥ 3-nt in length) to each end of the RNA target.
11. Use RNAfold^15^ to ensure that addition of flanking regions does not cause formation of stable secondary structures.

**Figure 7:**
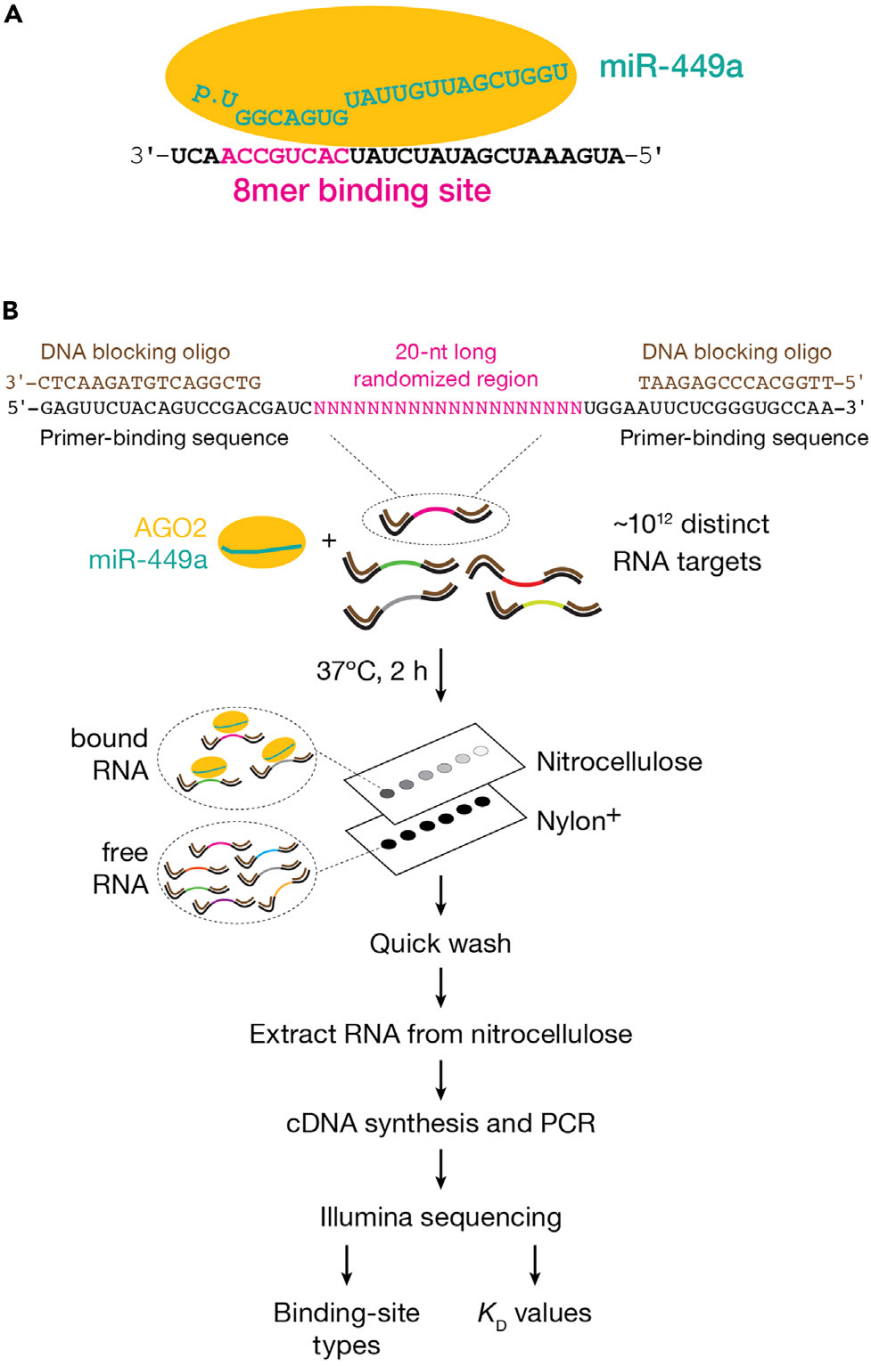
Overview of RISC binding and RBNS (A) Canonical 8mer site: contiguous pairing to the miRNA nucleotides 2–8, and an A opposite miRNA nucleotide 1. (B) RBNS consists in incubating RISC with a large pool of distinct RNA molecules. After reaching binding equilibrium, the reaction is applied to nitrocellulose and nylon membranes to separate RISC-bound RNA molecules from unbound RNA. Molecules retained on the nitrocellulose filter are used for Illumina library preparation and are sequenced. Analysis of sequencing reads identifies binding motifs and estimates *K*_D_ values.
13. Radiolabel the RNA target (steps 1−4).
14. Dilute the target RNA in Dilution Buffer to 2.5 nM.
15. Dilute RISC in Storage Buffer to 0.015, 0.03, 0.06, 0.08, 0.1, 0.15, 0.3, 0.6, 0.8, 1, 1.5, 2, 3, and 4 nM.
16. Set up 5-μL binding reactions by mixing 4 μL diluted RISC and 1 μL of 2.5 nM target RNA. Include a no-RISC binding reaction (4 μL Storage Buffer mixed with 1 μL of 2.5 nM target RNA).

***Note:*** Final concentration of target RNA in the binding reactions is 1 μL × 2.5 nM / 5 μL = 0.5 nM. Final concentrations of RISC are 4 μL / 5 μL ×(0.015, 0.03, 0.06, 0.08, 0.1, 0.15, 0.3, 0.6, 0.8, 1, 1.5, 2, 3, and 4) nM.

***Note:*** RNA targets with AU-rich flanking regions are typically less structured than RNA targets with GC-rich flanking regions. E.g., a 28-nt target RNA with a miR-449a 8mer binding site, a 3-nt 5′ flanking region, and a 17-nt 3′ flanking region: 5′-AUG AAA UCG AUA UCU AUC ACU GCC AAC U (Figure 7A).

12. Purchase the RNA with normal desalting.

17. Incubate the binding reactions at 37°C for 1 h.
18. Separate RISC-bound RNA from unbound RNA by using Bio-Dot apparatus (steps 6−8). Upon binding (step 7a), apply 4.5 μL of each reaction to the membranes. Wash with 10 μL ice-cold Wash Buffer (step 7c).
19. Expose the membranes to a storage phosphor imaging screen for 2−16 h (step 8h).
20. Measure the fraction of active RISC (Quantification and statistical analysis: step 1).

### Measuring binding affinity by double filter-binding assays

**Timing: 5−6 days**

We describe below the specific steps for performing double-filter binding assays to measure the binding affinity of a target RNA of interest.

***Note:*** To measure *K*_D_, we vary protein concentration, while keeping the concentration of target RNA constant. Importantly, the “titration” regime—in which the concentration of a binding site is much greater than *K*_D_—must be avoided (reviewed in ref.^16^). *K*_D_ can be determined under experimental conditions, in which the concentration of target RNA should be well below or of the same order of magnitude as its *K*_D_. The latter regime requires fitting the binding data to an appropriate binding equation that explicitly accounts for bound protein and does not rely on the common assumption that [Protein]_free_ ∼ [Protein]_total_.

21. Design a target RNA with a sequence complementary to the binding site of interest.

***Note:*** Mammalian AGO2 cleaves extensively complementary RNA targets at the phosphodiester bond opposite guide positions 10 and 11. To block cleavage, target RNAs extensively complementary to miRNA guides are designed with a phosphorothioate linkage flanked by 2′-*O*-methyl ribose at positions opposite to miRNA nucleotides 10 and 11 (ref.^17^^,^^18^^,^^19^).

22. Add flanking regions (≥ 3-nt in length) to each end of the RNA target.
23. Use RNAfold^15^ to make sure that addition of flanking regions does not cause formation of stable secondary structures.
24. Purchase the RNA with normal desalting.
25. Radiolabel the RNA target (steps 1−4).
26. Set up the binding reactions in 5 μL as described in steps 14−16.

**CRITICAL:** Include a no-RISC binding reaction.

***Note:*** To measure *K*_D_ of medium or low affinity binding sites, we use 100 pM (f.c.) radiolabeled target RNA and vary RISC concentration between 1 pM and 400 pM (f.c.).

27. Incubate the binding reactions at 37°C for 1 h.

**CRITICAL:** Be sure to provide sufficient time for equilibration.

***Note:*** For the binding equilibrium where RISC interacts with an RNA site_*i*_, the equilibration rate constant is described by

$$k_{eq}=k_{on}\times \left[RISC\right]\times \left[{site}_{i}\right]+k_{off}\text{.}$$

Estimate the equilibration rate constant for the protein and the target RNA of interest. E.g., for the let-7a 6mer-A1 binding site (a target RNA with Watson-Crick pairing to miRNA nucleotides at positions 2−6 and an adenosine opposite miRNA nucleotide at position 1), $k_{on}$ = 2.0 ± 0.1 × 10^8^ M^−1^·s^−1^ and $k_{off}$ = 0.24 ± 0.001 s^−1^ (ref.^9^). For the let-7a non-canonical 11mer-m11.21 site (a target RNA with Watson-Crick pairing to miRNA nucleotides at positions 11−21), $k_{on}$ = 3.6 ± 0.1 × 10^7^ M^−1^·s^−1^ and $k_{off}$ = 0.79 ± 0.08 s^−1^ (ref.^9^). At target RNA concentration of 100 pM, the binding reaction with the lowest RISC concentration (1 pM) should reach equilibrium in 4 ± 1 s and 1.2 ± 0.2 s for 6mer-A1 and 11mer-m11.21, respectively.

28. Separate RISC-bound RNA from unbound RNA by using Bio-Dot apparatus (steps 6−8). Upon binding (step 7a), apply 4.5 μL of each reaction to the membranes. Wash with 10 μL ice-cold Wash Buffer (step 7c).
29. Expose the membranes to a storage phosphor imaging screen for 2−16 h (step 8h).
30. Measure the binding affinity (Quantification and statistical analysis: step 2).

### Measuring binding affinity by RNA bind-n-Seq (RBNS)

**Timing: 17−21 days**

We describe below the specific steps for performing double-filter binding assays to measure the binding affinities of many binding sites simultaneously using the high-throughput sequencing method RBNS.

***Note:*** As in double filter-binding assays, the RNA pool concentration is the same for all binding reactions, while the protein concentration varies. As discussed in ref.,^1^ protein and RNA pool concentrations should be carefully chosen to avoid the “titration” regime. In a typical RBNS experiment, the random sequence RNA region is flanked by constant primer-binding regions used for sequencing. This design simplifies library preparation, avoids biases that can result from RNA ligation, and ensures that any RNA carried over from protein purification will not contaminate the sequenced library. The length of the random sequence region of RNA ligands is an important aspect of RBNS design and has been discussed previously.^20^ We randomized 20 nucleotides, obtaining an RNA pool of 4^20^ = 1.1 × 10^12^ distinct RNA sequences. Figure 7B illustrates the method. In addition to simultaneously determining absolute *K*_D_ values of various binding sites, our computational procedure estimates the concentration of active RISC^1^; therefore, measuring the fraction of active RISC by titration is not strictly required.

31. Purchase RNA targets (100 nmol) for RBNS as a pool of RNA oligonucleotides, 5′-phosphorylated GAG UUC UAC AGU CCG ACG AUC NNN NNN NNN NNN NNN NNN NNU GGA AUU CUC GGG UGC CAA, synthesized with equal ratio of bases (25:25:25:25) selecting the hand-mixed option for randomizing bases and PAGE purification.

***Note:*** Alternatively, RNA oligonucleotides can be purchased with normal desalting and purified on 8% polyacrylamide urea gels.

***Note:*** Constant primer-binding sequences may bias the RBNS assay if they contain a motif for protein binding.^1^ Constant regions cannot be readily modified to avoid biases, as they must remain compatible with Illumina sequencing. Inspired by,^21^ we use cDNA oligonucleotides to block the common sequences present in each RNA molecule, leaving only the randomized sequence and four 5′ and 3′ flanking nucleotides accessible for RISC binding. Moreover, making these regions double-stranded disfavors intramolecular secondary structures.

32. Purchase cDNA oligonucleotides (Table 1) with normal desalting: 5′-end and 3′-end blocking cDNA oligonucleotides, reverse transcription (RT) primer, PCR Forward primer, and Multiplexing PCR Reverse primer.
33. Radiolabel RNA input pool (steps 1−4).
34. Anneal cDNA blocking oligonucleotides to RNA input pool.
  a. Mix the following reagents:

**Table:** 

5′-Phosphorylated, radioactively labeled RNA input pool (4 μM)	5.0 μL
Reaction Mix Buffer (10×)	12.0 μL
5′-end blocking cDNA oligonucleotide (2 μM)	12.5 μL
3′-end blocking cDNA oligonucleotide (2 μM)	12.5 μL
Nuclease-free water	67.7 μL
Total	109.7 μL

    ***Note:*** The table shows the volumes to perform ten binding reactions. Linearly scale-up accordingly to the number of samples.
  b. Incubate the reaction mix 1 min at 95°C, 10 min at 65°C and 10 min at room temperature (20°C−25°C).
35. Set up binding reactions.
  ***Note:*** Each experiment typically includes five binding reactions. The highest concentration of RISC used corresponds to 40% (v/v) of the stock solution and equals 0.8–5 nM (f.c.) active protein. For additional reactions, we dilute the stock serially 3.2-fold in Storage Buffer. Each experiment also includes a no-RISC control containing protein Storage Buffer only.
  a. Prepare the reaction mix by adding to the RNA input pool from the previous step:
    i. 2.1 μL 0.2 M magnesium acetate,
    ii. 1 μL 2 mg·mL^−1^ tRNA,
    iii. 5 μL 40 U·μL^−1^ RNasin Plus RNase inhibitor,
    iv. 1.2 μL 1% (w/v) CHAPS,
    v. 1 μL 10 μg·μL^−1^ ultrapure BSA.
      ***Note:*** This amount of reaction mix is sufficient to perform 10 binding reactions. Linearly scale-up accordingly to the number of samples.
      **CRITICAL:** Even small amounts of RNA non-specifically retained on the nitrocellulose filter may dilute protein-bound RNA and reduce the sensitivity of RBNS. Therefore, tRNA and BSA are included to minimize non-specific binding. Although they do not interact with RISC or target RNA, BSA does interact with the nitrocellulose filter and can reduce its binding capacity.
  b. Pipet reaction mix into six 200-μL sterile PCR tubes on ice, 12 μL per tube.
  c. Dilute RISC in ice-cold Storage Buffer.

**Table:** 

Dilution ID	Dilution factor	[RISC]_active_	RISC	Storage Buffer
D1	1	1,320 pM	8.5 μL of stock	0 μL
D2	3.2	413 pM	3 μL of stock	6.58 μL
D3	10.2	129 pM	1.5 μL of stock	13.8 μL
D4	32.8	40 pM	3 μL of D3	6.58 μL
D5	105	13 pM	1.5 μL of D3	13.8 μL
  d. Add 8 μL diluted RISC to PCR tubes containing 12 μL reaction mix to generate a 20-μL reaction. For the no-RISC control reaction, add 8 μL Storage Buffer to 12 μL reaction mix.
    ***Note:*** The final concentration of the RNA input pool is 100 nM; the concentrations of individual binding sites in the pool should be measured by high-throughput sequencing.
  e. Incubate reactions for 2 h at 37°C.
36. Separate RISC-bound RNA from unbound RNA by using Bio-Dot apparatus (steps 6−8). Upon binding (step 7a), apply 19.5 μL of each reaction to the membranes. Wash with 100 μL ice-cold Wash Buffer (step 7c).
37. Expose the membranes to a storage phosphor imaging screen for 10 s to 15 min (step 8h).
38. Measure the fraction bound (Quantification and statistical analysis: steps 1a–b).

***Note:*** Imaging both nitrocellulose and nylon membranes at this step enables estimating the fraction of RNA input pool bound by RISC. Fraction bound is not used in downstream computational analyses of RBNS data. Nevertheless, we highly recommend performing this step as it helps in troubleshooting potential technical issues before proceeding with the time-consuming steps of RNA extraction, library preparation and high-throughput sequencing. Unbound RNA is not sequenced; therefore, the nylon membrane can be discarded once imaged.

39. Extract RISC-bound RNA from nitrocellulose membrane.
  a. Excise sample spots from the nitrocellulose membrane with a clean razor blade by following pencil mark drawn in step 6i.
  b. Fold each piece of membrane using forceps and put it in a new 1.7-mL tube. Do not touch the sample spots with the forceps.
  c. Prepare a proteinase K master mix by mixing the following components:

**Table:** 

Proteinase K Buffer (2×)	2 mL
Proteinase K (20 μg·μL^−1^)	200 μL
Glycogen (20 μg·μL^−1^)	10 μL
Nuclease-free water	1,790 μL
Total	4 mL

    ***Note:*** The table shows the volumes to perform ten reactions. Linearly scale-up accordingly to the number of samples. Because the expected amount of RNA is low, we add 1 μL 20 μg·μL^−1^ glycogen to each sample to maximize RNA recovery during the ethanol precipitation in step 39i.
  d. To each tube, add 400 μL the proteinase K master mix.
  e. Immerse pieces of nitrocellulose membrane into the solution by gently pushing with a sterile pipette tip.
  f. To 2 pmol of 5′-phosphorylated, radioactively labeled RNA input pool (i.e., the substrate in the binding reactions), add 400 μL of the proteinase K master mix.
    ***Note:*** RNA input pool is sequenced along with protein-bound RNA samples and is used in the downstream computation analyses to account for any sequence biases present in the random region of the substrate RNA. The RNA input pool needs to be sequenced only once; it is not necessary to perform this step for every experiment. However, the sequence composition may differ between independently synthesized pools, so each new RNA input pool must be sequenced.
  g. Incubate for 1 h at 45°C shaking at 300 rpm.
  h. Purify RNA by phenol/chloroform extraction as in steps 4b−g.
    ***Note:*** The chloroform step (steps 4e–g) is optional.
  i. Precipitate the RNA by adding three volumes of ethanol.
    ***Note:*** The RNA solution contains 150 mM NaCl from the Proteinase K Buffer, so there is no need to add 0.1 volumes 3 M sodium acetate.
  j. Incubate the solution 1 h on ice or overnight (12–18 h) at −20°C.
  k. Recover the RNA as in steps 1g−n.
    ***Note:*** Instead of vortexing, mix gently by inverting the tubes.
  l. Dissolve pellets in 9.1 μL nuclease-free water and proceed immediately to the next step.
    ***Note:*** Alternatively, RNA may be stored at −20°C by strictly following institutional guidelines for storage of radioactive material.
40. Reverse transcribe RNA to produce cDNA suitable for library amplification.
  a. Transfer 9 μL of each sample to a 200-μL PCR tube.
  b. Incubate 1 min at 90°C and 5 min at 65°C to denature any secondary structure.
  c. Add 1 μL 50 μM RT primer.
  d. Anneal the RT primer to RNA by incubating 5 min at 65°C and 10 min at room temperature (20°C−25°C).
  e. Place tubes on ice.
  f. Perform reverse transcription in a 20 μL final volume by adding to annealed RNA and RT primer from the previous step:
    i. 4 μL 5× First-Strand Buffer,
    ii. 1 μL 100 mM DTT,
    iii. 3 μL dNTP mix (10 nM each),
    iv. 2 μL RT Superscript III (200 U·μL^–1^).
  g. Mix well and incubate at 55°C for 1 h then 70°C for 15 min in a thermal cycler. Place tubes on ice.
    ***Note:*** Alternatively, store cDNA at –20°C until ready to perform the next step.
41. Removal of RNA template and precipitation of cDNA products:
  a. Transfer cDNA from the previous step to a new 1.7-mL tube.
  b. To hydrolyze template RNA in the sample after reverse transcription, add 180 μL of 0.44 M NaOH (f.c. 0.4 M).
  c. Incubate for 1 h at 55°C shaking at 500 rpm.
  d. Precipitate cDNA by adding 0.1 volumes 3 M sodium acetate (pH 5.2), 1 μL of 20 μg·μL^−1^ glycogen and 2.5 volumes of ethanol.
  e. Incubate the solution 1 h on ice or overnight (12–18 h) at −20°C.
  f. Spin at the maximum speed for 30 min at 4°C in a microcentrifuge.
  g. Carefully remove nearly all supernatant.
  h. Add 0.5 mL ice-cold 70% ethanol to the cDNA pellet and mix gently by inverting the tubes.
  i. Spin at the maximum speed for 15 min at 4°C in a microcentrifuge.
  j. Carefully remove nearly all supernatant.
  k. Repeat the steps 41h–j.
  l. Spin at the maximum speed for 15 s at 4°C in a microcentrifuge to collect the residual ethanol on the wall of the tube and discard the liquid using an extended pipette tip.
  m. Air dry the pellet for 5−20 min.
  n. Resuspend the pellet in 10.1 μL nuclease-free water.
  o. Store the samples at −80°C until ready to perform the next steps.
42. PCR amplification of libraries.
  ***Note:*** After reverse transcription, libraries are amplified by PCR using a universal primer and a barcoded primer to allow sample multiplexing. Primer sequences are listed in Table 1. The number of amplification cycles required to amplify the libraries should be minimized to avoid overamplification, which increases PCR artifacts. To determine the appropriate number of PCR cycles to use, the experimentalist may perform a test PCR amplification as described.^22^ We generally perform 16 PCR cycles.
  a. Setup PCR reactions in a final volume of 25 μL by combining:
    i. 10 μL water,
    ii. 2.5 μL 10× AccuPrime Pfx Reaction mix,
    iii. 1 μL 10 μM PCR Forward primer,
    iv. 1 μL 10 μM Multiplexing PCR Reverse Primer PCRIdx (x stands for the barcode number),
    v. 0.5 μL 2.5 U·μL^−1^ AccuPrime Pfx DNA Polymerase,
    vi. 10 μL cDNA (from step 41n).
  b. Place the PCR tubes in a thermocycler with the heated lid set to 105°C and perform PCR amplification using the following cycling parameters:

**Table:** 

Steps	Temperature	Time	Cycles
Initial denaturation	95°C	2 min	1
Denaturation	95°C	15 s	16
Annealing	65°C	30 s	
Extension	68°C	15 s	
Hold	4°C	forever
43. Gel-purify PCR products.
a. Resolve PCR products on a 2% (w/v) agarose gel in 1× TAE containing 0.5 μL·mL^−1^ (f.c.) ethidium bromide and place the gel on a clean sheet protector.
  **CRITICAL:** Skip a well between samples to prevent cross-contamination.
b. View the gel on a UV transilluminator and excise gel fragments between ∼120 and ∼160 bp with a clean razor blade.
  ***Note:*** Expected size of PCR amplicons is 138 bp (Figure 8).

44. Extract DNA using MinElute Gel Extraction kit according to the manufacturer’s instructions. Elute DNA in 10 μL of nuclease-free water.

**Figure 8:**
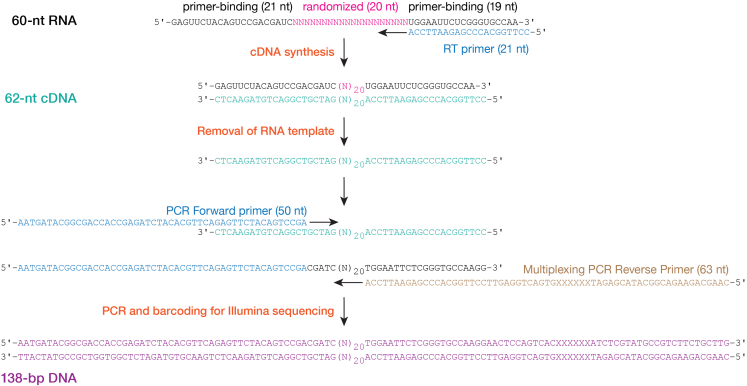
Steps of generating RBNS libraries for Illumina sequencing
45. Run 1 μL purified DNA libraries (0.1–10 ng) on an Agilent 2100 Bioanalyzer High Sensitivity instrument using the High Sensitivity DNA Kit following the manufacturer’s instructions.
46. Quantify concentration of libraries using Kapa Library Quantification Kit following the manufacturer’s instructions.
47. Pool libraries to be sequenced in an equimolar ratio to yield 1 nM (f.c.).

***Note:*** Library pooling can be performed by using the “Illumina Pooling Calculator”.

**CRITICAL:** we use a NextSeq-500 instrument and the NextSeq 500/550 High Output Kit v2.5 (75 Cycles) (Single-read sequencing). This system uses two-channel sequencing chemistry. To correctly identify DNA clusters and perform accurate base calling, all four DNA bases must be represented in every cycle. RBNS libraries show low diversity: RNA oligonucleotides of the input pool used in RBNS reactions are the same length (60 nt) and contain the same 20 nt 5′ sequence, among which five last nucleotides are sequenced. To provide the required cycle-to-cycle diversity, we add to the library pool 2−3 libraries (∼160−180 bp) originating from a different application (e.g., mouse small RNA-Seq). Ideally, these libraries should represent 10−20% of the total number of sequencing reads.

48. Sequence pooled RBNS libraries using an Illumina Sequencing platform compatible with TruSeq Small RNA protocol following the manufacturer’s instructions.

***Note:*** we use a NextSeq-500 instrument and the NextSeq 500/550 High Output Kit v2.5 (75 Cycles) (Single-read sequencing). Sequencing 14−16 pooled libraries in one run typically yields 12–25 million sequenced reads per library or 10−20 million sequenced reads after filtering and provides enough coverage for de novo site discovery and estimation of *K*_D_ values for sequence motifs ≤ 10 nt within a 20-nt random region.

49. Measure binding affinity for binding sites of interest as described in Quantification and statistical analysis: step 3.

## Expected outcomes

### Measuring the fraction of active RISC

**Figure 9:**
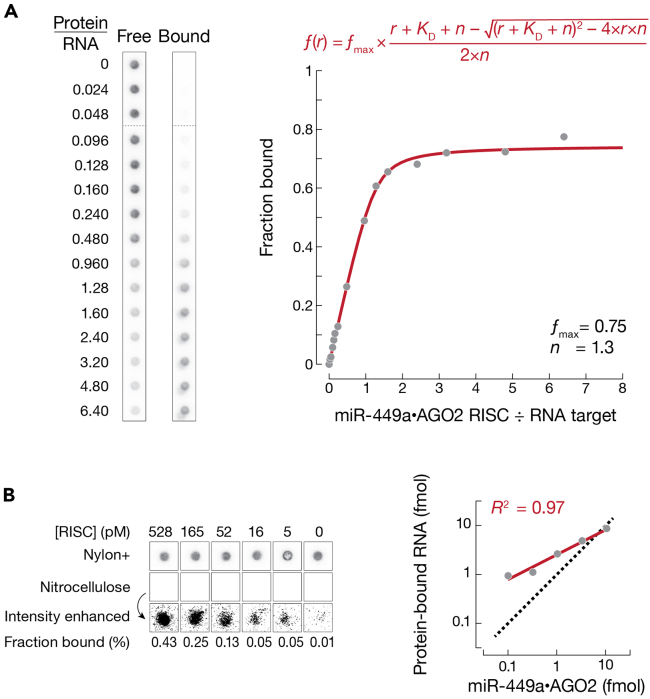
Measuring fraction bound by double filter-binding assays (A) Measuring the concentration of binding-competent RISC using a titration experiment. *K*_D_ is the apparent dissociation constant, r is the molar ratio of [RISC] to [target RNA], n is the stoichiometric equivalence point, ƒ is the fraction bound, and $f_{\max}$ is the maximum fraction bound. (B) After the binding step in RBNS, the two membranes are separated, imaged, and quantified. The fraction bound enables calculating the amount of protein-bound RNA, which is compared to the expected amount assuming 1:1 stoichiometry of RISC:target RNA complex (dashed line).

The nitrocellulose filter preferentially retains protein and protein-bound RNA. The positively charged nylon filter beneath the nitrocellulose traps protein-free RNA not retained by the nitrocellulose. Equilibrium binding is measured in a “titration regime”, i.e., the concentration of RNA target is much greater than its known *K*_D_. Essentially all added RISC binds to target RNA (the first linear portion of the curve in Figure 9A), until there is no more free target RNA left to bind (plateau of the curve in Figure 9A). Assuming that the stoichiometry of the bound complex is known, the breakpoint in fraction bound versus the ratio of protein to ligand indicates the amount of active protein. One AGO2 RISC molecule binds one molecule of target RNA^19^; therefore, in Figure 9A, the ratio of 1.3 suggests that miR-449a·AGO2 preparation is 77% active (1 / 1.3 = 0.77).

### Measuring RISC binding affinity by double filter-binding assays

To measure *K*_D_, the concentration of target RNA should be well below or of the same order of magnitude as its *K*_D_. The latter regime requires fitting the binding data to an appropriate binding equation that explicitly accounts for bound protein. Therefore, the *K*_D_ value is obtained by fitting the data to the Morrison quadratic equation. An example of assessing *K*_D_ values of let-7a·AGO2 by a double filter-binding assay can be found in figure 6C of ref.^19^

### Measuring binding affinity by RBNS

As protein concentration increases, the amount of RNA bound to the nitrocellulose filter increases (Figure 9B). Assuming (1) the stoichiometry of the bound complex RISC:target RNA is 1:1 and (2) complete recovery of protein from the nitrocellulose filter, the amount of protein-bound RNA should not exceed the amount of RISC in each binding reaction. Nevertheless, the amount of protein-bound RNA is typically > 1 (the dashed line in Figure 9B), especially at low RISC concentrations, because RNA non-specifically retained on the nitrocellulose filter dilutes the small amount of specifically recovered RNA.

Selection of bound RNA using nitrocellulose filter is based on the fact that nucleic acids do not bind the nitrocellulose filter and pass through, while many proteins display a strong affinity for the nitrocellulose filter without losing their affinity for RNA ligands. Nevertheless, some protein-free nucleic acids, mostly G-rich sequences, bind to nitrocellulose in the absence of protein.^23^ A no-protein control reaction enables detecting and correcting for this non-specifically recovered RNA. In our experimental setup, stretches of guanine and cytosine nucleotides (e.g., GGGGGGGGGG and ACCCCCCCCC) are among the most enriched motifs.

**Figure 10:**
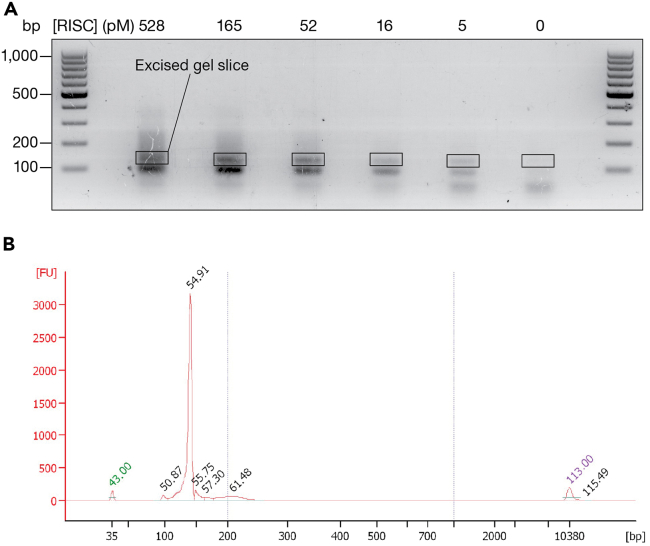
Purification and quality control of RBNS sequencing libraries (A) After the binding step, protein-bound RNA is extracted, reverse transcribed and amplified by PCR. PCR products of the correct size (138 bp) are gel-purified. (B) Size distribution of purified libraries is analyzed using an Agilent Bioanalyzer to control for the absence of residual PCR primers before sequencing.

During library preparation, recovered RNA is reverse transcribed and amplified by PCR. PCR amplicons, visualized on an agarose gel, correspond to three types of molecules of different sizes (Figure 10A): (1) PCR Forward and Multiplexing PCR Reverse primers have the lowest size (< 100 bp), (2) amplicons containing one sequencing adapter are ∼100-bp long, and (3) amplicons containing both sequencing adapters are 138-bp long. Therefore, PCR products of the correct size (138 bp) should be gel-purified. The size distribution of the purified libraries is then analyzed using an Agilent Bioanalyzer; this step enables controlling for the absence of residual PCR primers before quantifying and sequencing. Figure 10B provides an example of Agilent Bioanalyzer data.

Library preparation starting with protein-bound RNA resulting from 20-μL binding reactions comprising ∼5−1,000 pM RISC and 100 nM RNA input pool yields ∼10−200 ng of library as estimated by KAPA library quantification.

## Quantification and statistical analysis

1. Measuring the fraction of active RISC by titration.
  a. Use software (e.g., ImageJ or ImageQuant TL) to quantify signal of protein-bound ($S_{bound}$) and unbound RNA ($S_{unbound}$) from each binding reaction on nitrocellulose and nylon membranes, respectively, as well as background (b) of the membranes.
    **CRITICAL:** Be sure to define regions of interest of the same area for all measurements.
  b. For each binding reaction, compute the fraction bound f as:
    $$f=\frac{S_{bound}-b}{\left(S_{bound}-b\right)+\left(S_{unbound}-b\right)}$$
  c. Subtract the fraction bound of no-RISC binding reaction from each titration point to correct for non-specific RNA retention.
  d. Plot the fraction bound f as a function of RISC concentration to target RNA concentration (r=([RISC])/([targetRNA])).
  e. Fit the titration data to the quadratic form of the Hill equation^24^:
    $$f\left(r\right)=f_{\max}\times \frac{r+K_{D}+n-\sqrt{{\left(r+K_{D}+n\right)}^{2}-4\times r\times n}}{2\times n}\text{,}$$
    where $K_{D}$ is the apparent dissociation constant, n is the stoichiometric equivalence point, and $f_{\max}$ is the maximum fraction bound.
2. Measuring binding affinity by double filter-binding assays.
  a. Quantify the fraction bound f as described in steps 1a−c.
  b. Fit the binding data to the Morrison equation for tight binding:
    $$f\left(\left[E_{T}\right]\right)=\frac{\left(\left[E_{T}\right]+\left[S_{T}\right]+K_{D}\right)-\sqrt{{\left(\left[E_{T}\right]+\left[S_{T}\right]+K_{D}\right)}^{2}-4\times \left[E_{T}\right]\times \left[S_{T}\right]}}{2\times \left[S_{T}\right]}\text{,}$$
    where $\left[E_{T}\right]$ is total enzyme concentration, $\left[S_{T}\right]$ is total target RNA concentration, and $K_{D}$ is the apparent dissociation constant.
3. Measuring binding affinity by RBNS.
  a. Download demultiplexed samples from the sequencing instrument.
  b. Select Illumina reads containing TGG (the first nucleotides of the 3′ adapter) at positions 21−23.
  c. Remove the 3′ adapter sequence (5′-TGG AAT TCT CGG GTG CCA AGG).
  d. Filter sequencing reads by removing those with Phred quality score < 20 for all nucleotides and containing “N” base calls.
  e. Convert the 20-nt remaining sequences to RNA by replacing thymine nucleotides with uracil.
  f. To the 5′ and 3′ ends of the 20-nt central region, append the constant flanking sequences that were accessible in binding reactions.
    ***Note:*** If cDNA blocking oligonucleotides were not used in the experiment, append GAG UUC UAC AGU CCG ACG AUC to the 5′ end and UGG AAU UCU CGG GUG CCA A to the 3′ end of the 20-nt central region. If cDNA blocking oligonucleotides were annealed to the RNA input pool and only four nucleotides of constant primer-binding sequence on either side were single-stranded, append GAUC to the 5′ end and UGGA to the 3′ end of the 20-nt central region.
  g. Interrogate each sequencing read from the previous step in RNA input pool and RISC-bound libraries for presence of binding sites of interest.
    ***Note:*** We assign a read to a site category if it contains a single binding motif. Reads containing multiple instances of binding sites (from the same or a different site category) and reads containing partially overlapping sites are discarded. Reads that do not have any binding motifs of interest are classified as reads with no-site.
  h. To estimate *K*_D_ values, run the code by following instructions in the README file.

## Limitations

The method presented here has broad utility in quantitatively assessing the specificity of RNA- or DNA-binding proteins for nucleic acids. Purified proteins are a prerequisite for binding assays. Contaminants carried over from protein purification may bind target RNA molecules and affect *K*_D_ estimation. We assume that RISC binds one target RNA molecule with 1:1 stoichiometry and a Hill coefficient of 1 and that the recovery of bound RNA is complete. For *K*_D_ estimation, we also assume that the reaction has reached equilibrium. Upon separating protein-bound RNA from unbound RNA using Bio-Dot apparatus and washing the filters with a buffer without reactants, the forward reaction rate is zero and the products start to dissociate to achieve a new equilibrium. Therefore, we wash rapidly once, and we assume that equilibrium is unperturbed. To the extent possible, we recommend confirming binding affinities by an additional, independent assay (e.g., a kinetic experiment). To measure *K*_D_, the “titration” regime—in which the concentration of target RNA is much greater than *K*_D_—must be avoided. Therefore, when the binding affinity is completely unknown, a series of pilot experiments is required to determine the correct range of concentrations of protein and ligand. *K*_D_ values can be fit to motifs of interest if at least 10–100 sequencing reads are assigned to these binding sites.

## Troubleshooting

### Problem 1

No filtration is observed in some wells of Bio-Dot apparatus (related to steps 13b−c).

### Potential solution

• If sample volume exceeds 100 μL, load 8−10 samples at a time and close the other wells with tape to increase the pressure per area.
• If there is a well with no filtration, close the other wells with tape and remove potential bubbles obstructing the filtration by pipetting the liquid up and down with a tip.

### Problem 2

Bio-Dot apparatus does not assemble well and/or leaks between wells (related to steps 12−13).

### Potential solution

• Nylon and nitrocellulose membranes should not extend beyond the edge of the gasket. Membranes of a larger size may obstruct vacuum formation after the Bio-Dot apparatus has been assembled. Trim the membranes if needed.
• Leakage between wells may be due to incomplete tightening of the apparatus. Once the Bio-Dot apparatus has been assembled, adjust screw tightening using a diagonal crossing pattern while under gentle vacuum.

### Problem 3

No signal of protein-bound RNA is detected on nitrocellulose membranes (related to step 14f).

### Potential solutions

• Measure concentration of binding-competent protein (steps 15−23) to ensure that the protein has not lost its activity.
• Perform a positive control using a high-affinity substrate. If the problem persists, a double filter-binding assay may not be suitable for the protein of interest. For example, detecting binding of fly Dicer to pre-miRNAs by double-filter assay yields poor results, whereas mobility shift experiments with the same protein preparation readily detect binding.

### Problem 4

Signal of unbound RNA on the nylon filter is saturated, preventing calculation of fraction bound (related to step 14f).

### Potential solution

• Unbound RNA retained on the nylon filter may be greater than protein-bound RNA retained on the nitrocellulose filter, especially in RBNS experiments. If exposed to a phosphorimaging screen for the same duration, signal of protein-bound RNA is barely detectable, while signal of unbound RNA is already saturated. In this case, nylon and nitrocellulose filters should be imaged twice: with a short and a long exposure times. Prepare five 5−10-fold serial dilutions of radioactively labeled RNA target and spot them onto a separate nylon filter; these samples constitute imaging standards. Imaging nylon and nitrocellulose filters together with the imaging standards at two different time points enable quantifying both signals of protein-bound and unbound RNA and estimating fraction bound.

### Problem 5

Library preparation in RBNS experiments yield small amounts of DNA, insufficient for Illumina sequencing (related to steps 45−46).

### Potential solutions

• Measure concentration of binding-competent protein (steps 15−23) to ensure that protein used in binding reactions has not lost its activity.
• RISC can bind a variety of sites with affinities ranging from ∼1 pM to ∼10 nM. If the protein of interest binds substrates with lower affinities, we recommend performing the binding reaction in a larger volume and using higher protein concentrations.
• We also recommend ethanol precipitating the RNA and cDNA (steps 39h−i and 41d, respectively) 1 h on ice or overnight (12–18 h) at −20°C to increase recovery.
• Finally, the number of PCR cycles in step 42b may be increased. However, it is important to minimize the number of amplification cycles used to avoid overamplification, which increases PCR artifacts.

### Problem 6

PCR amplified sequencing libraries contain molecules migrating slower than DNA of the excepted size (related to electrophoresis in step 43).

### Potential solutions

• In most cases, this phenomenon is caused by over-amplification of the libraries. In later amplification cycles, the desired library-primer hybridization may be outcompeted by library-library hybridization. The resulting annealing products are called “PCR-bubbles” and are partly double-stranded (hybridized adapters) and partly single-stranded (noncomplementary inserts). Libraries showing bubble products can be sequenced, as bubble products will be denatured into single-stranded DNA prior to flow cell binding. Nevertheless, formation of bubble products may yield in partial loss of PCR amplicons during the size selection step. To avoid PCR-bubbles, reduce the amount of cDNA input or the number of PCR cycles in step 42b.

## Resource availability

### Lead contact

Further information and requests for resources and reagents should be directed to and will be fulfilled by the lead contact, Phillip D. Zamore (phillip.zamore@umassmed.edu).

### Materials availability

This study did not generate new unique reagents.

### Data and code availability

This study did not generate new datasets or code.
